# Evaluation of physical health status beyond daily step count using a wearable activity sensor

**DOI:** 10.1038/s41746-022-00696-5

**Published:** 2022-11-09

**Authors:** Zheng Xu, Nicole Zahradka, Seyvonne Ip, Amir Koneshloo, Ryan T. Roemmich, Sameep Sehgal, Kristin B. Highland, Peter C. Searson

**Affiliations:** 1 Measurement Corps, In Health, Johns Hopkins University School of Medicine, Baltimore, MA USA; 2 Institute of Nanobiotechnology, Johns Hopkins University, Baltimore, MA USA; 3 Center for Movement Studies, Kennedy Krieger Institute, Baltimore, MA USA; 4 Department of Physical Medicine and Rehabilitation, Johns Hopkins University School of Medicine, Baltimore, MA USA; 5 Respiratory Institute, Cleveland Clinic, Cleveland, OH USA; 6 Department of Biomedical Engineering, Johns Hopkins University, Baltimore, MA USA; 7 Department of Materials Science & Engineering, Johns Hopkins University, Baltimore, MA USA

**Keywords:** Preclinical research, Biomedical engineering

## Abstract

Physical health status defines an individual’s ability to perform normal activities of daily living and is usually assessed in clinical settings by questionnaires and/or by validated tests, e.g. timed walk tests. These measurements have relatively low information content and are usually limited in frequency. Wearable sensors, such as activity monitors, enable remote measurement of parameters associated with physical activity but have not been widely explored beyond measurement of daily step count. Here we report on results from a cohort of 22 individuals with Pulmonary Arterial Hypertension (PAH) who were provided with a Fitbit activity monitor (Fitbit Charge HR^®^) between two clinic visits (18.4 ± 12.2 weeks). At each clinical visit, a maximum of 26 measurements were recorded (19 categorical and 7 continuous). From analysis of the minute-to-minute step rate and heart rate we derive several metrics associated with physical activity and cardiovascular function. These metrics are used to identify subgroups within the cohort and to compare to clinical parameters. Several Fitbit metrics are strongly correlated to continuous clinical parameters. Using a thresholding approach, we show that many Fitbit metrics result in statistically significant differences in clinical parameters between subgroups, including those associated with physical status, cardiovascular function, pulmonary function, as well as biomarkers from blood tests. These results highlight the fact that daily step count is only one of many metrics that can be derived from activity monitors.

## Introduction

Wearable activity sensors enable remote monitoring of an individual’s physical activity, but have been largely limited to assessment of average daily step count. Walking, or ambulating, is a fundamental movement of daily life and has become an important metric in promoting human health^1^. For example, increasing daily step count (from <4000 to ≥12,000) is associated with a decrease in all-cause mortality^2,3^. In hospitalized patients, daily step count thresholds (typically < 1000 steps per day) have been associated with poor outcomes, such as readmissions^4–6^. Related ambulation parameters, such as gait speed^7–9^ and timed walk tests^10,11^, have also been found to be predictive of clinically relevant outcomes.

Historically, remote monitoring of an individual’s physical status has been challenging, however, advances in wearable technology have enabled continuous assessment following surgery, or between clinic visits for patients with chronic diseases. Wearable inertial measurement units (IMUs), such as Fitbit devices, record step count along with other metrics derived from the IMU signals (e.g. sleep) that can be viewed in the associated smart phone app. In addition, many wearable devices, such as Fitbit, use photoplethysmography to measure heart rate.

Step count, and particularly daily step count, remains the most common metric for remote assessment of physical activity, however, minute-to-minute step count and heart rate data can be downloaded from the Fitbit server using their application programming interface (API). Therefore, for an individual who wears a Fibit continuously, 10,080 values of step rate (units: steps per minute, SPM) and heart rate (units: beats per minute, BPM) can be obtained over one week, each point representing the average value of step rate and heart rate for that minute. While the accuracy of step count measurements in free-living settings and in patient populations with atypical gait patterns remains a concern^12,13^, studies in individuals with cancer, cardiovascular disease, pulmonary arterial hypertension, and multiple sclerosis suggest that these devices can provide accurate and clinically relevant data^14–17^. Similarly, in comparison studies, heart rate measurements from Fitbit devices general show good agreement with electrocardiograms for individuals at rest or at low activity levels^18,19^. However, other factors such as skin pigmentation may also influence measurement accuracy^20^.

The objective of this study was to show that clinically-relevant metrics, beyond daily step count, can be derived from wearable activity monitors. There is a large resource of untapped information contained within the data from these devices, enabling a much more granular fingerprint of an individual’s activities of daily life. Here we present results from analysis of 22 individuals with Pulmonary Arterial Hypertension (PAH) who were each provided with a Fitbit device between two clinic visits. From minute-to-minute step rate and heart rate data we derived a range of parameters associated with physical activity during free-living, including metrics associated with the weekly heart rate and step rate distributions, parameters related to the intensity, length, and frequency of ambulations, an analog of the Physical Working Capacity test to assess fitness, a free-living 6-minute walk distance (FL6MWD), as well as weekly usage metrics. We also considered a metric of health state based on comparison of the FL6MWD to predicted values for healthy individuals with the same age, gender, and BMI as the subject. Principal Component Analysis and Latent Profile Analysis were used to identify subgroups of patients based on Fitbit metrics.

At the clinic visits a maximum of 26 measurements were recorded (7 continuous and 19 categorical), including assessment of health related quality of life (HRQOL) and WHO Functional Class, the presence or absence of various symptoms, and assessments of organ function. Continuous variables included heart rate measurements, six minute walk test (6MWT), right ventricular systolic pressure (RVSP), and three biomarkers from blood tests. To assess the potential for clinical relevance, we used a thresholding approach to compare clinical parameters between the two subgroups. We show that many Fitbit-derived metrics can be used to identify subgroups with differences in clinical parameters associated with physical status, cardiovascular function, pulmonary function, as well as biomarkers from blood tests. In addition, we show that several of the Fitbit metrics were strongly correlated with continuous clinical parameters. Overall, we show that Fitbit-derived metrics can provide insight into an individual’s activities of daily life, and have the potential to support decision making and clinical care.

## Results

### Heart rate and step rate

**Fig. 1:**
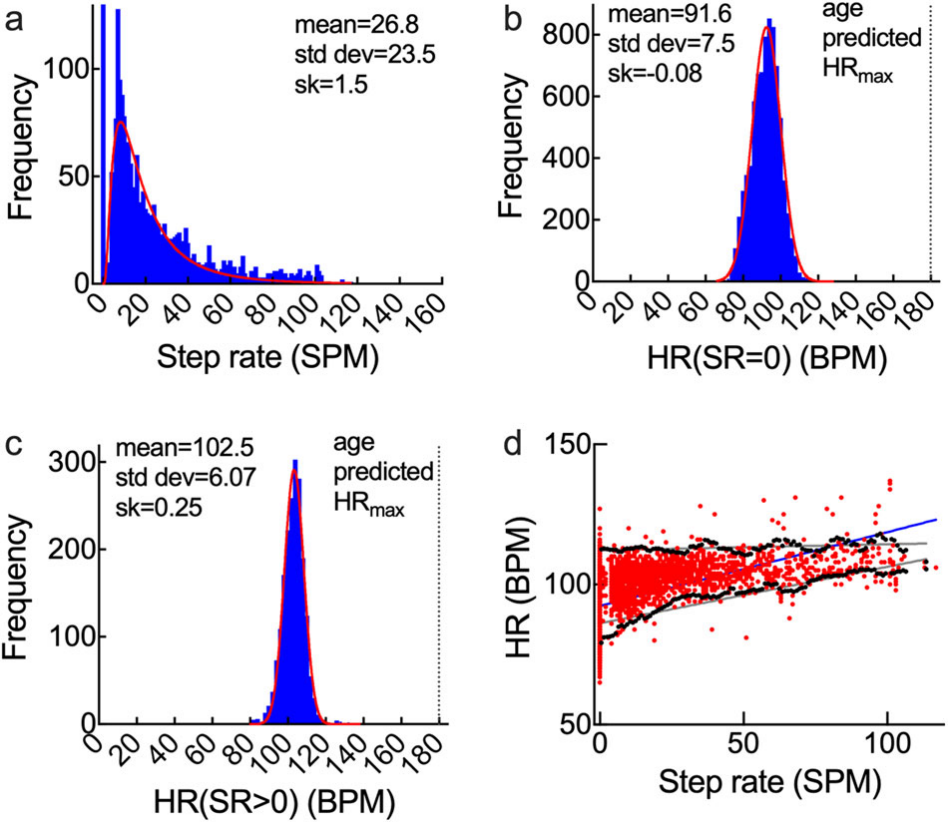
Representative weekly heart rate and step rate (PAH 14/week 11). **a** Distribution of minute-to-minute step rate (SR) (units: steps per minute, SPM). Red line shows a log normal fit. **b** Distribution of heart rate (BPM) for all minutes where SR = 0. Red line shows a normal fit. **c** Distribution of heart rate (BPM) for all minutes where SR > 0. Red line shows a normal fit. **d** Weekly activity map: scatter plot showing heart rate versus step rate. Each point represents one minute where a physiological heart rate was recorded. The grey lines show the upper and lower envelopes of the activity map. The blue line shows a linear least squares fit to the data.

We analyzed 3.5 × 10^6^ min of Fitbit data for 22 individuals with PAH between two outpatient clinic visits with an average of 18.4 ± 12.2 weeks for each subject (total 405 weeks). The average age of the subjects was 50.6 ± 13.4 years old (mean ± SD), with 3 males and 19 females. In most cases, data were analyzed in weekly blocks from Sunday to Saturday. From the weekly data we obtained a maximum of 10,080 values (100% usage) of step rate (units: steps per minute, SPM) and heart rate (units: beats per minute, BPM). The distribution of step rates (SR) over each week (excluding SR = 0) typically followed a log normal distribution (Fig. 1a), from which we extracted mean, standard deviation, and skewness. For these subjects, the maximum step rate was typically around 100 SPM, spanning medium walking (80–99 SPM) and brisk walking (100–119 SPM)^21^. For active healthy individuals additional peaks are usually observed in the step rate histograms in the 100–120 SPM range, corresponding to brisk walking, and in the 150–180 SPM range, corresponding to running^21^. We note that cadence analysis is widely used to assess exercise intensity in free living environments in a wide range of populations^21–23^. The means of the weekly step rate histograms for each subject were 13.6–32.8 SPM, with a standard deviation in the range 10.2–30.3 SPM. The skewness varied from 1.19–2.55, showing that there was a moderate to large increase in density to the right of the most probable value. As a comparison to the commonly used daily step count, the average daily step count was in the range 1338–10,679 steps, with an average of 5729 steps per day (Supplementary Fig. 1).

To assess the potential for clinical significance, we used a thresholding approach. We first divided the subjects into two groups based on a threshold value, and then compared the 26 clinical parameters (Supplementary Table 1) between groups. We compared individuals with average daily step counts >5000 steps (14/22) to those with <5000 steps (8/22). This arbitrary threshold resulted in 6 statistically significant clinical parameters (Supplementary Table 2 and Supplementary Fig. 2). Subjects with <5000 steps per day had lower 6MWD at visit 1, lower hemoglobin levels at visit 2, poorer pulmonary health (higher physician-assessed WHO FC) at visit 1, and experienced more pedal edema (Pedal Edema score) at visit 2. Two subjects had average daily step counts >10,000 steps per day (PAH 1, 19), but had no other similarities. Sensitivity analysis of threshold values and the number of statistically significant clinical parameters for all Fitbit metrics are provided in Supplementary Figs. 3 and 4.

The minute-to-minute heart rate data for each week were separated into heart rate at SR = 0 (HR(SR = 0), i.e. no physical activity) and heart rate at SR > 0 (HR(SR = 0), i.e. active). Histograms for HR(SR = 0) (Fig. 1b) and HR(SR > 0) (Fig. 1c) were described by normal distributions, from which we obtained the mean, standard deviation, and skewness. The range of mean HR(SR = 0) was 66.2–111.8 BPM, with standard deviations of 6.4–13.7 BPM (Supplementary Fig. 5). The skewness varied from −0.75 to 2.30, highlighting a broad range of behavior with relatively large tails to the left and right of the peak (Supplementary Figs. 6 and 7).

The distribution of HR(SR = 0) represents all occurrences of zero step rate and may represent various postures under a range of resting conditions (e.g. transient or sustained). To relate HR(SR = 0) to resting heart rate (RHR), we considered two conditions: the mean value and the average of the lowest 10 values of HR(SR = 0). A previous study found that individuals with PAH with RHR below 82 BPM had significantly longer overall event-free survival during a median follow-up period of 37 (18–64) months^24^. In the study, the RHR was measured at a clinic visit during a stable period of at least 15 min of recumbent rest, but is likely higher than the true resting heart rate (see below). We compared subjects with mean values of HR(SR = 0) <82 BPM (14/22) to those with >82 BPM (8/22). This resulted in 8 statistically significant clinical parameters (Supplementary Table 2 and Supplementary Fig. 8). Subjects with lower mean values of HR(SR = 0) had lower RHR at visits 1 and 2, and lower peak heart rate at visit 2, but experienced more pedal edema (Pedal Edema score) and more palpitations (Palpitation score) at visit 1, were less able to perform usual activities (lower EQ-5D Usual Activity scores) at visit 1, and experienced more pain/discomfort (lower EQ-5D pain/discomfort scores) at visit 1.

Two subjects (PAH 1,4) had a mean HR(SR = 0) >100 BPM. Both subjects had low fitness slopes (see below), suggesting that they did not access a wide range of heart rate during daily activities. However, PAH 1 had the highest average daily step count in the dataset. We note that 3 subjects (PAH 4, 20, 27) removed the device overnight (see below), which may have resulted in higher mean HR(SR = 0) values since heart rate values during sleeping were likely not included.

The true resting heart rate (RHR) is usually defined as the value obtained in a supine position immediately after waking but before getting out of bed^25^. To obtain a value similar to the true RHR we calculated the average of the lowest 10 values of HR(SR = 0); we assumed that the 10 lowest values recorded during each week are most likely obtained while supine and resting for an extended period. The range of the average lowest weekly HR values was 50.3–69.2 BPM (Supplementary Fig. 5). A recent study of >90,000 individuals over 35 weeks, reported that the RHR (assumed to be the true RHR) was dependent on age, BMI and sleep duration, with daily values of RHR from 40–108 BPM^25^, although 95% of men and women had RHR values between 50–80 BPM, similar to the range found here.

We compared individuals with skewness of HR(SR = 0) <1 (11/22) to those with skewness >1. This resulted in 4 statistically significant clinical parameters (Supplementary Table 2 and Supplementary Fig. 9). Subjects with lower skewness values were more likely to have higher resting heart rate at visits 1 and 2, experienced less pain/discomfort (lower EQ-5D pain/discomfort scores) at visit 1 and were more likely to be in better health (higher EQ-5D Index) at visit 1. Two subjects had skewness of HR(SR = 0) values >1.9 (PAH 27, 28): both subjects also had relatively low resting heart rates, longer free-living 6MWD, and higher fitness plot slopes.

The heart rate at SR > 0 represents HR values while subjects were active. The mean values of HR(SR > 0) were 78.6–121.0 BPM (mean 94.4 BPM), and the standard deviation was 6.5–14.0 BPM (Supplementary Fig. 10). The mean values were only slightly higher than the mean values of HR(SR = 0), although the standard deviations were similar. The mean skewness values for HR(SR > 0) were from −0.57 to 1.35, similar to the range for HR(SR = 0). We compared individuals with mean values of HR(SR > 0) <95 BPM (12/22) to those with >95 BPM, resulting in 4 statistically significant clinical parameters (Supplementary Table 2 and Supplementary Fig. 11). Subjects with lower mean values of HR(SR > 0) had lower RHR at visits 1 and 2, lower albumin levels at visit 1, and experienced more palpitations (lower Palpitation score) at visit 1.

All of the weekly data can be represented on a scatter plot which maps out an individual’s weekly activity on a minute-to-minute basis (Fig. 1d). These activity maps are approximately triangular with relatively high density of points at low step rates and decreasing density as the step rate increases. Other parameters derived from the minute-to-minute data included assessment of fitness based on the Physical Working Capacity 170 (PWC 170) test, metrics associated with weekly ambulation, free-living 6-minute walk distance (FL6MWD), and device usage.

### Principle Component Analysis (PCA)

**Fig. 2:**
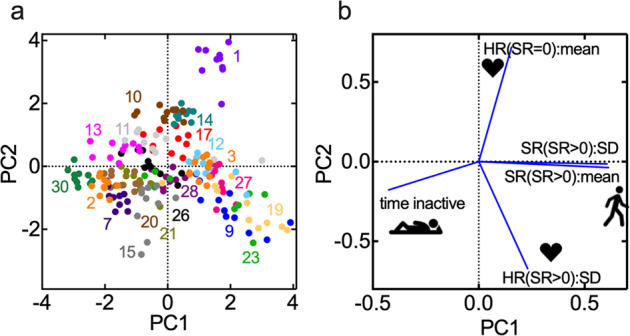
Principal component analysis. **a** PCA scatter plot of the first two principal components. For each subject (*N* = 20), 10 weeks were randomly selected from the dataset. The variance for the first two principal components were 48.6% and 30.0%, respectively. For 100 independent runs, the mean variance of PC1 and PC2 was 77.5 ± 0.58%. Numbers represent subject IDs. Each point represents one week of data. **b** Loading plot. HR(SR = 0):mean is the mean value of the heart rate at SR = 0; HR(SR > 0):SD is the standard deviation of the heart rate at SR > 0; SR(SR > 0):mean is the mean step count for SR>0; SR(SR>0):SD is the standard deviation of the step rate for SR > 0; time active is the fraction of minutes with SR = 0.

To identify subgroups within the subject population we performed principal component analysis (PCA). Five parameters were selected for analysis: the mean and standard deviation of the step rate histogram, the standard deviation of HR at SR > 0, the mean HR at SR = 0, and the fraction of time inactive (Fig. 2a). The data points for each week for most subjects were tightly clustered in distinct regions. From the loading plot (Fig. 2b), PC1 is dominated by the step rate parameters (+PC1) and the fraction of time inactive (−PC1). PC2 is dominated by the mean heart rate at SR = 0 (+PC2) and the standard deviation of the heart rate for SR > 0 (−PC2). The group of subjects in the fourth quadrant (PAH 3, 9, 12, 19, 23, 27) are characterized by high mean and standard deviation of the step rate, and a high value of the standard deviation of the heart rate at SR > 0. This implies that these individuals exhibit a wide range of step rates and a wide range of heart rates during normal activities of daily life. The group of subjects along the positive y-axis (PAH 1, 10, 14, 17) are characterized by high mean heart rate at SR = 0. High values of HR(SR = 0) imply that these individuals have a high resting heart rate and are unlikely to access a wide range of heart rates during normal activities, even if they have the capacity for moderate or high step rates. The group of subjects along the negative x-axis (PAH 2, 7, 11, 13, 20, 21, 30) are characterized by a large fraction of time inactive. Three subjects (PAH 15, 26, 28) are clustered around the origin. The PCA plot suggests a range of behavior with distinct combinations of metrics associated with heart rate and step rate. To explore these relationships in more detail, we assessed several derived parameters. Distinct groupings of subjects were found for mean HR(SR = 0) >82 BPM, skewness of HR(SR = 0) <1, ambulation product, *P* > 1000, and fitness slope >0.15 (Supplementary Fig. 12).

### Fitness

**Fig. 3:**
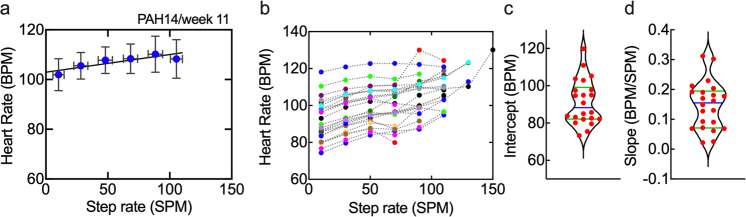
Assessment of fitness. **a** The average step rate and corresponding average heart rate in 20 SPM bins for PAH 14 in week 11. Bars represent mean ± SD. **b** Mean step rate versus mean heart rate plots averaged over all weeks for each individual. **c** Fitness intercept (BPM) and **d** fitness slope (BPM/SPM) obtained from a linear least-squares fit to the HR-SR plots for each individual. Blue bar–mean; green bars–upper and lower quartiles.

To derive a metric related to physical fitness from the Fitbit data we determined the mean step rate, in 20 SPM bins, and the mean heart rate in that bin. Assuming that the step rate is related to power output, this approach is similar to the Physical Working Capacity PWC170 protocol, where the slope of a power vs. heart rate curve is used to predict the power output at 170 BPM as a measure of fitness. An example from a weekly data set shows that the mean heart rate generally increases with increasing step rate (Fig. 3a). It is also evident that the extrapolated heart rate at SR = 0 is relatively high, about 103 BPM. The fitness plot averaged over all weeks of data was almost identical to the example week (Fig. 3b), showing that there was only small weekly variation. The fitness plots for all subjects (Fig. 3b) spanned a wide range, with intercepts from 73–120 BPM (Fig. 3c) and slopes of 0.02–0.31 BPM/SPM (average = 0.15 BPM/SPM) (Fig. 3d). In general, individuals with a low heart rate at SR = 0 accessed a broader range of heart rates with increasing activity level (step rate) and hence had higher slopes.

Comparison of subjects with fitness slope >0.15 (11/22) to those with slope <0.15, resulted in 3 statistically significant clinical parameters (Supplementary Table 2 and Supplementary Fig. 13). Notably, subjects with slopes >0.15 had lower NT-proBNP levels at visits 1 and 2. B-type natriuretic peptide (BNP) and N-terminal pro b-type natriuretic peptide (NT-proBNP) are biomarkers for cardiac stress, and PAH patients with NT-proBNP levels below about 300 pg L^−1^ are considered low risk for heart failure^26^. The mean levels for subjects with slope >0.15 at visits 1 and 2 were 188 ± 180 and 145 ± 165 pg mL^−1^, respectively. These results suggest that the fitness slope may be a useful indicator of NT-proBNP levels and risk for heart failure. Comparison of subjects with fitness intercepts above (10/22) and below (12/22) the mean (91 BPM) were similar to results for subgroups with HR(SR = 0) above and below 95 BPM.

### Ambulation metrics: endurance, intensity, frequency

**Fig. 4:**
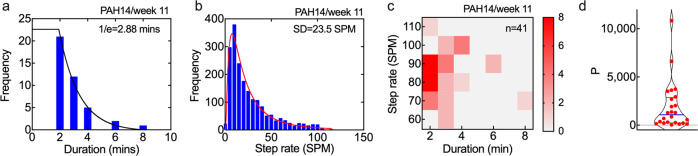
Ambulation metrics. Representative ambulation metrics for PAH 14 in week 11. **a** Frequency of weekly ambulations of ≥2 min with sustained SR ≥ 60 SPM. Endurance was defined by the 1/e value of an exponential fit to the histogram. **b** Ambulation intensity was defined by the standard deviation of the step rate distribution. **c** Heat map showing ambulation frequency on a plot of step rate versus ambulation duration. **d** Violin plot showing average values of ambulation product *P* (=frequency × endurance × intensity) for all subjects. Blue bar–mean; green bars–upper and lower quartiles.

We defined an ambulation as a period of at least 2 min with SR ≥ 60 SPM, which corresponds to slow walking or faster^23^. A 2-minute duration was selected since the 2-minute walk test (2MWT) is widely used to assess functional capacity^27,28^. From the number of weekly ambulations we obtained metrics for frequency, endurance, and intensity. The average ambulation frequency for all subjects was in the range 1.6–96 ambulations per week. A measure of a subject’s endurance was obtained from the 1/e value of an exponential fit to the histogram of weekly ambulation durations (Fig. 4a). Therefore, individuals with longer duration ambulations would be considered to have greater endurance. Endurance (1/e) values were between 2.2–7.0 min. The longest individual ambulation duration was 70 min (PAH 9). A measure of ambulation intensity was obtained from the standard deviation of the weekly step rate histogram (Fig. 4b), i.e. how far above the mean value: values ranged from 10–30 SPM. In general, the ambulation frequency was higher for shorter durations and intermediate values of step rate (Fig. 4c).

To compare individuals, we defined the ambulation product *P* as frequency × endurance × intensity. Average values for all subjects varied from 42.8 to 10,845.3 (*P*_mean_ = 1910) (Fig. 4d). Comparison of subjects with *P* > 1000 (12/22) to those with *P* < 1000, resulted in 7 statistically significant clinical parameters (Supplementary Table 2 and Supplementary Fig. 14). An ambulation product value of 1000 was selected as it was close to the median value (1079), and represented a well-defined separation between the two groups (Fig. 4d). Subjects with *P* < 1000 had lower 6MWD at visits 1 and 2, and experienced more pedal edema (Pedal Edema score) at visit 1. Two subjects had ambulation product values > 5000 (PAH 9, 19). Both subjects had a high ambulation frequency and walked more than 5000 steps per day on average. Both subjects also had relatively lower resting heart rates, longer free-living 6MWD (see below), and higher fitness plot slopes. PAH 1, despite having the highest step count, ranked fourth in ambulation product value as a result of having relatively lower endurance and intensity values.

### Device usage

Usability and compliance with usage are critically important in deploying devices in free-living settings. Since a measured heart rate (i.e. 20 ≤ HR ≤ age predicted maximum heart rate) implies that the device is worn, we defined usage as the fraction of minutes during a week with a physiological heart rate value (i.e. minutes worn/10,080). The fraction of minutes outside this range was very small (4.2 × 10^–7^%), and hence in most cases it may be reasonable to use all recorded data points (i.e. HR > 0) for analysis. In this study the average weekly usage was 0.44–0.97. Note that charging the device overnight (e.g. 8 h) once a week results in a weekly usage of 0.95. We also defined the maximum off-time as the longest continuous time during the week that the device was not worn, which varied from less than 1 h to more than 12 h. From heat maps of usage and the maximum off-times for all subjects (Supplementary Figs. 15 and 16) we can further infer how the device was used.

**Fig. 5:**
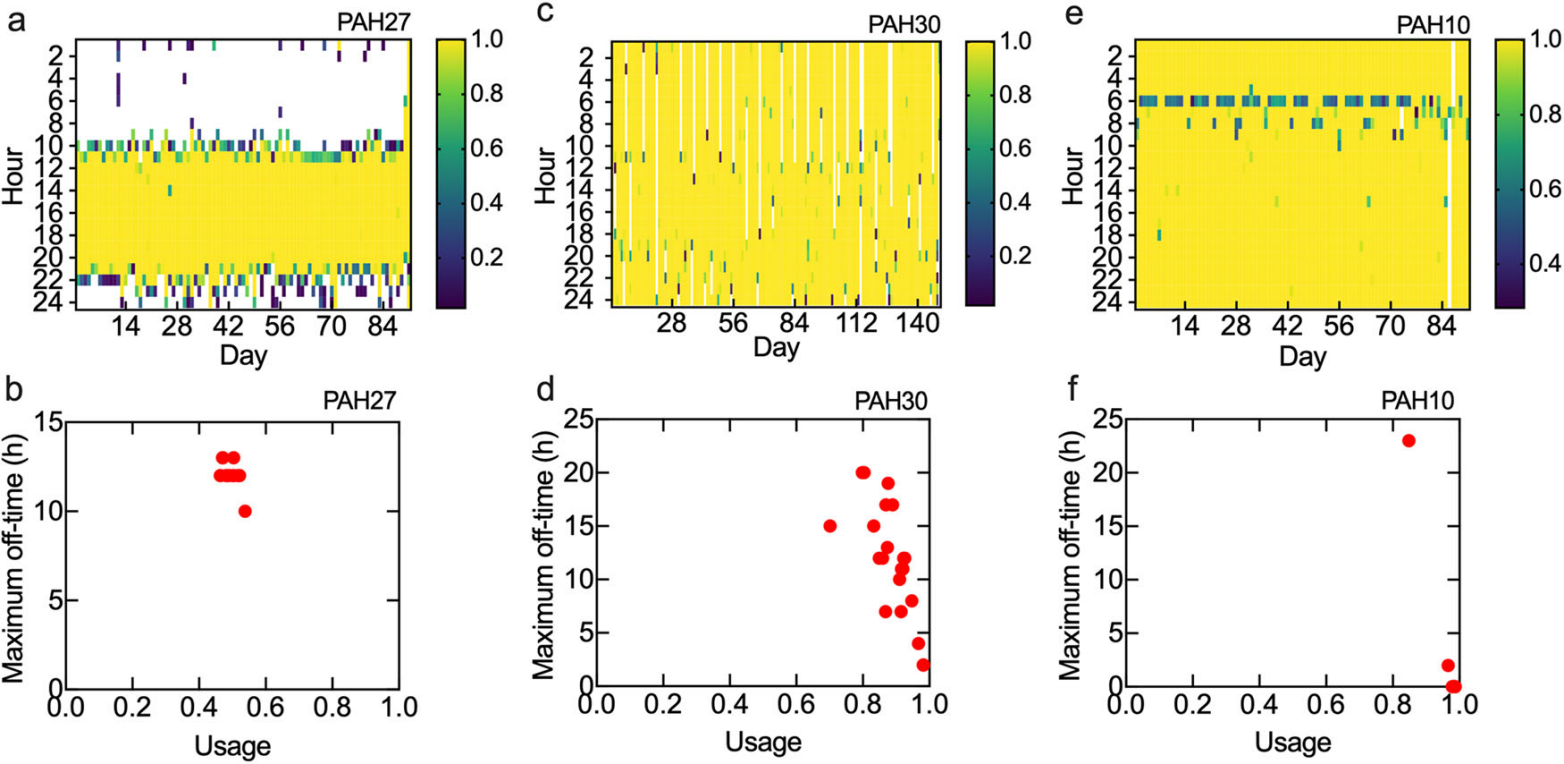
Examples of heat maps of device usage and maximum off time. Heat maps show usage for each hour of each day during the trial. The legend indicates the fraction of an hour that the device was worn with HR > 0. Yellow cells indicate that the device was worn continuously for the full hour. White cells indicate that the device was not worn (no HR recorded) for the full hour. **a** Heat map for PAH27 (13 weeks of data), showing low usage (average = 0.49) with the device not worn overnight. **b** The maximum off time for each week for PAH27 is consistently around 12 h overnight. Each point represents the maximum off-time for each week in the trial. **c** Heat map for PAH30 (22 weeks of data), showing relatively high usage (0.90), with the device removed for several hours every few days. **d** The maximum off time for PAH30 is typically 8–20 h and includes overnight hours. **e** Heat map for PAH10 (13 weeks of data), showing high usage (0.97). For the first 10 weeks the maximum off-time is less than 1 h. **f** The maximum off time for PAH10 is usually less than 1 h.

As an example, the usage heat map for PAH27 (average usage = 0.49) (Fig. 5a) shows that the device was consistently not worn overnight but was routinely worn during the day, resulting in a maximum off-time of around 12 h for each week (Fig. 5b). The heat map for PAH30 (average usage = 0.90) (Fig. 5c) shows that the device was not worn overnight approximately every 7 days, likely for overnight charging. The maximum off time (Fig. 5d) varied from 2–20 h, but for most weeks was 7–12 h, consistent with overnight charging. For PAH10 (average usage = 0.97) the heat map (Fig. 5e) indicates that the device was worn almost continuously except for 2 h in week 10 and 24 h in week 13 (Fig. 5f). The frequent partial usage hours from 06:00 to 07:00 suggest that the device was taken off to charge for less than one hour in the morning. Many subjects removed the device overnight approximately every 7 to 10 days, presumably for charging. Three subjects (PAH 14, 20, 27) consistently removed the device overnight. Three subjects (PAH 10, 19, 26) showed regular partial usage for about an hour, either in the morning or the evening, likely for charging.

From the distribution of average usage (Supplementary Fig. 17), 7 of 22 subjects had usage >0.94, which corresponds approximately to the 75th percentile. Comparison of usage, resulted in 4 statistically significant clinical parameters (Supplementary Table 2 and Supplementary Fig. 18). Subjects with average weekly usage < 0.94 (15/22) were more likely to have more severe PAH (higher EQ VAS score) at visit 1, worse pulmonary health (higher physician assessed WHO FC score) at visit 1, and experienced more difficulty breathing (modified Borg dyspnea score) at visit 2. Two subjects had average usage < 0.5 (PAH 4, 27), however, both of these subjects removed the device overnight. The third subject who removed the device overnight (PAH 20) also had low average usage (0.60). (Changes in device usage over time are summarized in Supplementary Figs. 19 and 20).

### Free-living 6-minute walk distance (FL6MWD)

The 6MWT is a sub-maximal exercise test used to assess aerobic capacity and endurance, and was introduced by the American Thoracic Society in 2002^29^. The 6MWT is widely used in different patient populations^30–32^, with thresholds for prediction of survival typically in the range 300–350 m for individuals with chronic respiratory diseases, including individuals with PAH^33^. Timed walk tests provide information on distance and speed^10,11^, but are labor intensive and hence are impractical to implement at high frequency during hospitalization or outside the clinic. At the research level, apps for self-administered 6MWTs have been tested with wearable accelerometers^34^. We have developed a novel approach for assessment of a free-living 6MWD (FL6MWD) by searching the weekly data for the continuous 6-min block of time with the highest cumulative step count. Step count was converted to distance based on a subject’s gender and height (Supplementary Figs. 21 and 22).

**Fig. 6:**
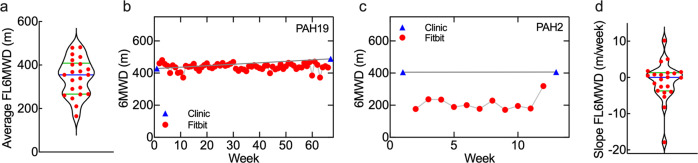
Clinic 6MWD and FL6MWD. **a** Violin plot showing distribution of average values of FL6MWD for all subjects. Mean = 344 m. Blue bar–mean; green bars–upper and lower quartiles. **b** Example of a subject with good agreement between FL6MWD and interpolated clinic values (group 1, “performer”). (Triangles) clinic values, (circles) FL6MWD. The grey line shows the linear interpolation of the clinic values. **c** Example of a subject where the FL6MWD was lower than the interpolated clinic values (group 2, “underperformer”). (Triangles) clinic values, (circles) FL6MWD. The grey line shows the linear interpolation of the clinic values. **d** Violin plot of the slope of the FL6MWD (m/week) over time for all subjects. Blue bar–mean; green bars–upper and lower quartiles.

The average values of FL6MWD ranged from 164 m (PAH30) to 478 m (PAH23) (Fig. 6a). The lowest weekly FL6MWD was 85.7 m (PAH30, week 23) and the highest weekly value was 683.1 m (PAH23, week 11). 14/22 subjects had average values >320 m (PAH1, 3, 9, 10, 11, 12, 14, 17, 19, 22, 23, 26, 27, 28). Comparison of FL6MWD resulted in 6 statistically significant clinical parameters (Supplementary Table 2 and Supplementary Fig. 23). Notably, subjects with average FL6MWD < 320 m had lower 6MWD at visit 1 and visit 2, experienced more pedal edema (Pedal Edema score) at visit 2, had worse pulmonary health (higher physician-assessed WHO FC) at visit 1, and had lower hemoglobin at visit 2.

The lowest average FL6MWD was 164 m (PAH30). This subject was the second heaviest (108.7 kg), and had an ambulation *P* value < 1000. Two subjects had average FL6MWD values > 480 m (PAH3, 23). These subjects were in the fourth quadrant of the PCA plot, which implies that they had a wide range of step rates and heart rates during normal weekly activity, and had ambulation product *P* values > 1000.

### FL6MWD and therapeutic goals

As described above, 6MWD threshold values of around 320 m are predictive of poor survival in PAH patients. In the context of treatment goals, a 6MWD of ≥380–440 m has been proposed for PAH patients^35–37^. Subjects with average FL6MWD > 400 m (12/22) had higher 6MWD at visit 2, lower NTpro-BNP at visit 2, experienced less chest pain (Angina score) at visit 1, and had better pulmonary health (lower physician-assessed WHO FC) at visit 2 (Supplementary Table 2 and Supplementary Fig. 24).

### Comparison of clinic 6MWD to FL6MWD

From comparison of FL6MWD to the values measured at the two clinic visits (see *Methods* and Supplementary Fig. 25 for details), we identified two distinct groups of individuals. In one group (group 1: “performers”), we found excellent agreement between the FL6MWD and the interpolated value of the clinic 6MWD (Fig. 6b), whereas in the second group (group 2: “underperformers”), the FL6MWD was lower than the clinical values (Fig. 6c). Subjects in group 1 were older and shorter, had lower 6MWD at visits 1 and 2, and had fewer problems in walking (lower EQ-5D Mobility score) at visit 2 (Supplementary Fig. 26). These results suggest that the daily activity for the “underperformers” was below their physical capacity.

To assess changes over time during the trial, we determined the slope of the FL6MWD (see *Methods* and Supplementary Fig. 27 for details). Values ranged from −17.9 m/week to +10.2 m/week (mean + 1.0 ± 5.4 m/week) (Fig. 6d). The subject with the most negative slope (PAH23) initially maintained high values of FL6MWD for the first 13 weeks, but then recorded a much lower value in subsequent weeks (Supplementary Fig. 22), suggesting a significant change in lifestyle or health state. Three subjects had a large positive slope (>4.0 m/week) (PAH3, 10, 20), and four subjects had a large negative slope (<4.0 m/week) (PAH1, 13, 21, 23).

### Free-living 6MWD (FL6MWD) and physical health state (PHS)

**Fig. 7:**
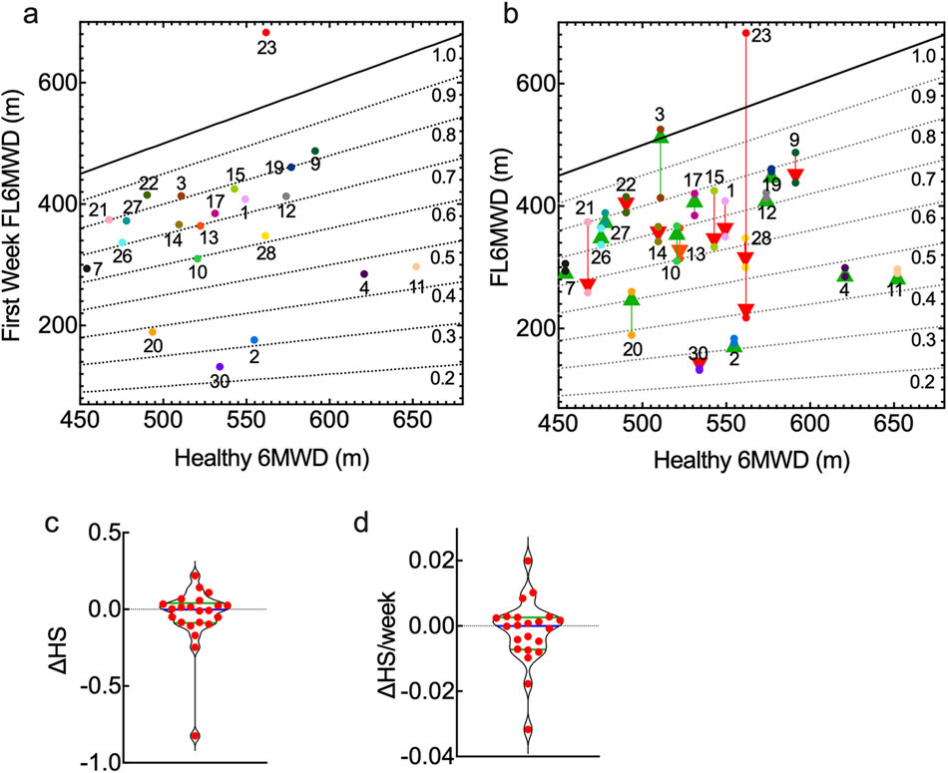
Free-living 6MWD versus the predicted 6MWD for a healthy individual (H6MWD) of the same age, gender, and BMI. **a** Data for all subjects in week 1. Each point represents the value of FLWD in week 1. The dotted lines represent constant values of physical health state (PHS = FL6MWD/H6MWD). Numbers represent subject IDs. **b** PHS in the first and last week. The values of FL6MWD in the last week were obtained from the value in week 1 and the slope of a linear fit to the weekly FL6MWD following smoothing. Numbers represent subject IDs. The arrows represent the change during the trial. Red arrows represent a decrease in PHS, green arrows represent an increase in PHS. **c** Violin plot showing the change in PHS during the trial. **d** Normalized change in PHS. Blue bar–mean; green bars–upper and lower quartiles.

To estimate a subject’s physical health state (PHS), we calculated the predicted 6MWD for an equivalent healthy individual (H6MWD) with the same age, gender, and BMI as the subject (see *Methods* for details). We defined a subject’s physical health state as the ratio of FL6MWD/H6MWD. A plot of the FL6MWD in week 1 versus the predicted H6MWD shows a wide range of values of PHS, from about 0.25 to more than 1 (Fig. 7a). 13/22 subjects had PHS values of about 0.7–0.8 (i.e. values of FL6MWD in the range of 70–80% of the equivalent healthy individual), 3 subjects had values around 0.6, and 5 subjects had values below 0.5. Subject 23 had a ratio > 1 but, as described previously, this subject recorded high FL6MWD values during the first 13 weeks, but then maintained a much lower value in subsequent weeks. It is evident that there is no correlation between the FL6MWD in week 1 and the predicted 6MWD (H6MWD) for an equivalent healthy individual (Fig. 7a).

We next assessed the change in PHS for each subject based on the values of FL6MWD in week 1 and in the last week prior to the second clinic visit (Fig. 7b). 11/22 subjects showed an increase in health state, and 11/22 subjects showed a decrease (Fig. 7c). The change in PHS was less than 10% for 15/22 subjects, and the normalized change in PHS was less that 1%/week for 18/22 subjects (Fig. 7d).

Two subjects showed an increase in PHS of more than 1%/week (PAH 3, 20), and two subjects showed a decrease of more than 1%/week (PAH 21, 23). Five subjects (PAH 3, 9, 19, 12, 27) maintained health state values greater than 0.72 for their first and last weeks. These subjects were in the fourth quadrant of the PCA plot with high mean and standard deviation of the step rate, and a high value of the standard deviation of the heart rate at SR > 0. Three subjects (PAH 30, 2, 20, 11) had health state values below 0.52 in their first and last weeks. These subjects were located along the negative x-axis of the PCA plot, characterized by a large fraction of time inactive.

### Comparison of Fitbit metrics

**Fig. 8:**
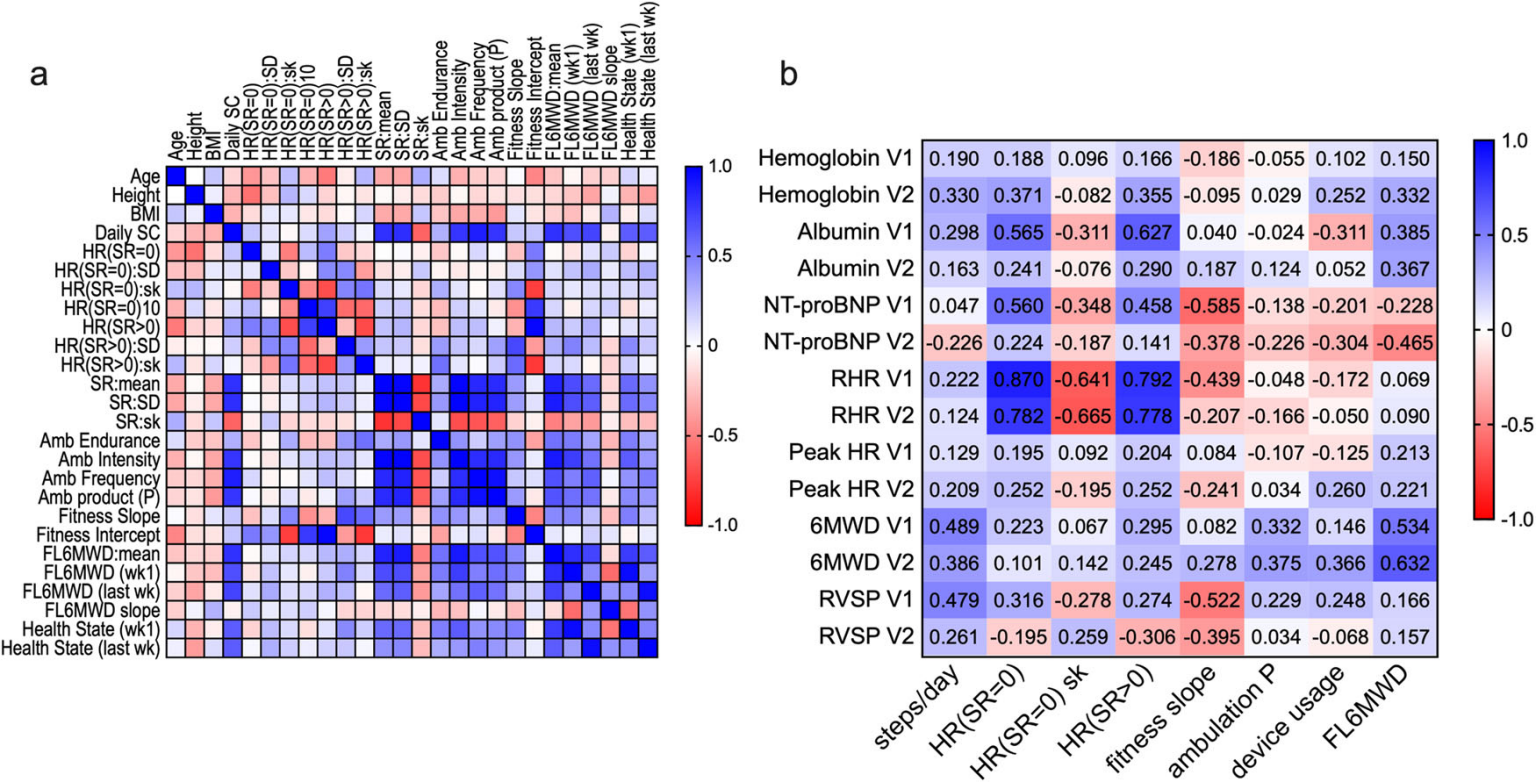
Correlation amongst Fitbit-derived parameters and between Fitbit-derived parameters and clinical parameters. **a** Correlation matrix (Pearson r value) of all the Fitbit-derived parameters and demographic variables (age, height, and BMI). **b** Correlation matrix (Pearson r value) for statistically significant continuous clinical parameters (y-axis) for subgroups identified from Fitbit parameters (x-axis).

Comparison of 23 Fitbit-derived metrics (Fig. 8a) revealed that relatively few of the heart rate parameters were strongly correlated with other parameters, suggesting that they measure diverse aspects of health status. Although several of the metrics derived from step rate were highly correlated to each other, there were also significant differences. For example, the mean weekly step rate was strongly correlated to three of the ambulation-related metrics but less well correlated to the FL6MWD metric. In addition, metrics such as the fitness slope were only weakly correlated to other metrics. The skewness of the step rate was strongly anticorrelated to the mean and standard deviation of the step rate, ambulation intensity, and ambulation frequency. This suggests that an increase in the mean weekly step rate introduces asymmetry into the distribution. This rich and diverse landscape suggests that the Fitbit metrics capture many different facets of health status.

To determine whether we could identify subgroups amongst the patients based on Fitbit metrics we performed Latent Profile Analysis. 8 Fitbit metrics were used as input: daily step count, HR(SR = 0), HR(SR = 0)sk, HR(SR > 0), ambulation *P* value, fitness slope. Based on the maximum Bayesian Information Criterion (BIC) (Supplementary Table 3), the subjects were categorized into three groups (Supplementary Fig. 28). Group 1 had high ambulation metrics (steps/day, ambulation product P, and FL6MWD), high HR(SR > 0), and high fitness slope (Supplementary Table 4). Group 2 were characterized by the lowest ambulation metrics (steps/day, ambulation product P, FL6MWD), the lowest HR(SR = 0) and HR(SR > 0), and the highest HR(SR = 0)sk. Group 3 had the highest HR(SR = 0) and HR(SR > 0), the lowest HR(SR = 0)sk and fitness slope. The three groups identified from LPA analysis occupied distinct regions of the PCA plot, with the exception of PAH 10 who was in Group 2 (Supplementary Fig. 29).

To further explore the relationship between Fitbit metrics and clinical parameters, we determined the correlation between 8 Fitbit metrics and the seven continuous clinical variables at the two clinic visits (see Fig. 8b). Five Fitbit metrics had strong correlations (Pearson *r* values > ±0.5). Albumin was correlated with HR(SR = 0) and HR(SR > 0) at visit 1 (*r* = 0.565 and 0.627, respectively). NT-proBNP was also correlated with HR(SR = 0) at visit 1 (*r* = 0.585), and was inversely correlated with fitness slope at visit 1 (*r* = −0.585). RHR at visits 1 and 2 were correlated with HR(SR = 0), HR(SR = 0)sk, and HR(SR > 0). 6MWD at visits 1 and 2 were correlated with FL6MWD. RVSP at visit 1 was inversely correlated with fitness slope. Notably, steps/day and ambulation P did not have strong correlations to the continuous clinical parameters.

## Discussion

Daily step count is a ubiquitous metric associated with wearable activity monitors. However, the underlying minute-to-minute heart rate and step rate measurements contain a rich array of data associated with physical and cardiovascular function and have the potential to provide much more detailed insight into an individual’s health status based on activities of daily living. In addition to metrics derived from the weekly distribution of minute-to-minute heart rate and step rate data, we derived several others, including: (1) an analog of the widely-used 6MWD, which we termed the free-living 6 min walk distance (FL6MWD), (2) an analog of the Physical Working Capacity test used to assess physical fitness, (3) metrics associated with the frequency, intensity, and duration of weekly ambulations, (4) metrics describing usage patterns, and (5) metrics associated with physical health status based on the FL6MWD and the predicted 6MWD for a healthy individual with the same gender, age, and BMI. These metrics are derived from a single wearable activity monitor during normal daily life and do not require any prescribed activities or external monitoring. Together these parameters provide weekly signature of an individual’s status that could be used for identifying subgroups within patient populations or assessing changes over time.

Principle component analysis showed that subjects could be grouped by parameters broadly associated with physical activity and cardiovascular function. Physical status was defined by step rate parameters (+x axis) or fraction of time inactive (−x axis). Cardiovascular status was defined by resting heart rate while inactive (+y axis) and the range of heart rates accessed during normal daily activities (−y axis). Subjects were clustered in different regions of the PCA plot both along the axes, indicating that their status was dominated by either physical or cardiovascular factors, or within a quadrant, indicating an influence of a combination of physical or cardiovascular factors. Latent Profile Analysis identified 3 groups located in distinct regions in the PCA plot, with characteristics consistent with the interpretation described above. For example, LPA Group 2 were located in the third quadrant in the PCA plot and were characterized by the lowest ambulation metrics and low heart rate metrics.

Device usage is an important factor in analyzing data from wearable devices. Using physiological values of heart rate as a proxy for a device being worn for each minute of a week enabled detailed analysis of usage patterns. Heat maps revealed a range of different usage patterns, such as removing the device overnight or wearing the device almost continuously. Usage patterns are also important in analyzing metrics related to activity. For example, daily step count may not be significantly influenced by removing the device overnight, whereas derived metrics such as fraction of time inactive may be subject to error. There are three key factors that contribute to the relatively high usage in this study: (1) the device is wrist worn and has a small form factor, (2) the battery life is up to 7–10 days, (3) many models are waterproof and hence do not need to be taken off between charges. Usage patterns and changes in usage patterns may contain additional information about an individual’s health state.

To establish the potential for clinical relevance, we used a thresholding approach to identify statistically significant differences between subgroups. Even within this relatively small population we found statistically significant differences in 18 parameters recorded at the initial or final clinic visits and based on 8 of the Fitbit-derived metrics. Clinical parameters included those associated with physical status, cardiovascular function, pulmonary function, as well as biomarkers from blood tests. The identification of statistically significant differences in a wide range of clinical parameters suggests that it may be possible to identify surrogate markers of disease severity in PAH. For example, ambulation product *P* < 1,000, and FL6MWD < 320 m were correlated with lower clinic measured 6MWD at visits 1 and 2. Subjects with fitness slope > 0.15 had lower NT-proBNP levels, an important biomarker of cardiac stress, at visits 1 and 2. In addition, this approach may contribute to identification of individuals who would benefit from more frequent clinic visits or specific medications.

Activity monitors can provide insight into an individual’s physical capacity in terms of activities of daily living. Many of these devices are relatively low cost and low maintenance following deployment. Long battery life and low form factor appear to be important in achieving high levels of compliance. Metrics derived from the raw data can be easily displayed in a dashboard and provide an additional tool for telemedicine.

In summary, we used the minute-to-minute step rate and heart rate data from a Fitbit device to derive a wide range of metrics associated with physical activity and cardiovascular function. These metrics were used to identify clusters of subjects with common characteristics. In addition, several Fitbit metrics were strongly correlated to continuous clinical parameters. Using a thresholding approach we showed that many Fitbit metrics resulted in statistically significant differences in clinical parameters between subgroups, including those associated with physical status, cardiovascular function, pulmonary function, as well as biomarkers from blood tests. These results highlight the fact that daily step count is only one of many metrics that can be derived from activity monitors. Importantly, this approach is generally applicable to remote monitoring of many patient populations.

There are several limitations in this study. (1) Although heart rate can be used to verify that a device is worn, independent validation of specific activities of daily life remains to be established. Ultimately, AI could be used to indicate the likelihood of specific types of activity. (2) Neither heart rate nor step rate measurements were independently validated. One approach to address this issue would be to perform independent measurements of resting heart rate while ambulating at fixed gait speed at the beginning of a trial. Independent laboratory studies of heart rate and step rate signatures in response to specific types of activities of daily life will also be important in refining data analysis. However, we note that comparison of Fitbit data to parameters from the two clinic visits (e.g. resting heart rate and 6MWD) provide support for the validity of the measurements. (3) In comparing Fitbit parameters to clinical parameters we did not consider factors such as a patient’s medication, or adjust for other covariates. Incorporating factors such as medications would likely improve the correlations to clinical parameters and provide insight into the role of these factors in activities of daily life, however, the sample size was too small. The threshold values used for comparison of clinical parameters were either guided by previous studies or selected arbitrarily. Studies in larger cohorts and in other patient populations will be essential to establishing the clinical relevance of this approach. (4) This is a small, single center study and the results remain to be reproduced in other patient populations. Nonetheless, these results show the potential for defining metrics beyond daily step count that can contribute to assessing an individual’s health status.

## Methods

### Study details

Data were obtained from a prospective, observational study where PAH patients wore a wrist worn activity monitor (Fitbit Charge HR^®^) between two outpatient visits. The study was approved by the IRB at the Cleveland Clinic (IRB number 15–1392). Participants provided written informed consent. Thirty subjects were enrolled in the study; two patients withdrew consent after enrollment. Study details have been published elsewhere^17^. Outpatient visits occurred during two consecutive routine appointments where patients received various tests. Overall, the clinical data included 19 categorical and 7 continuous variables. Categorical variables included eight scores from questionnaires associated with health related quality of life (HRQOL), modified Borg dsypnea score, and RV function scores, physician and patient assessed WHO Functional Class, and seven binary scores associated with presence or absence of a symptom, and six assessments of organ function. Continuous measurements included heart rate measurements, six minute walk distance (6MWD), right ventricular systolic pressure (RVSP), and three biomarkers from blood tests. A complete list of clinical parameters is provided in Supplementary Table 1.

### Analysis

For each subject the minute-to-minute heart rate and step rate data were obtained from the Fitbit server. 22 of 30 data sets were included for analysis: these data sets had clinical data for both visits and at least 4 weeks of Fitbit data between the two visits. 3 subjects were excluded because they had no data on the Fitbit server, 3 subjects did not have a second clinic visit, and 2 subjects had only 1 week of Fitbit data. Data were analyzed in weekly blocks from 00:00 on Sunday to 23:59 on Saturday. Therefore, depending on the day of the week of the clinic visits, there is a gap between the clinic visit and the first and last week of Fitbit data. Each data point represents the step rate (SPM) and average heart rate (BPM) over one minute. The device was considered worn if the average heart rate for any minute was greater than or equal to 20 BPM and less than or equal to the age predicted maximal heart rate (HR_max_(BPM) = 208–0.7 × age)^38^. The number weeks of Fitbit data between the two clinic visits was 18.4 ± 12.2 (range from 7 to 65 weeks). In total we analyzed 3.5 × 10^6^ min of data over 405 weeks. Overall, this corresponds to 85% of the total time.

### Statistical analysis

Baseline metrics from the week-long blocks of data were derived from the weekly values of step rate (SR), heart rate at step count equal zero (HR(SR = 0), i.e. no activity), and heart rate at step rate greater than zero (HR(SR > 0), i.e. active). From the distributions of these three metrics we obtained the mean, standard deviation, and the skewness. The heart rates were fit to a normal distribution, and the step rate was fit to a log normal distribution. A scatter plot of step rate versus heart rate provided a weekly signature of cardiovascular activity for each individual. From a linear least-squares fit to the data we obtained the slope (heart rate per step rate (BPM/SPM)). The effective area of the heart rate versus step rate (HR vs. SR) plot was determined by first calculating the upper (lower) envelopes. Each point in the upper and lower envelopes represents the average of the maximum (or minimum) HR values at each value of step count in a bin width of 10 SPM. The envelope point is located at the average step rate for all values with HR values. Step rates with no HR values are omitted from the calculation. Bins with no HR values do not have an envelope point. We then performed a linear least-squares fit to the envelopes to determine the area of the HR-SC plot.

The skewness of the step rate and heart rate distributions were obtained from:

1 $$Sk = \frac{{\mathop {\sum }\nolimits_{i = 1}^N \left( {x_i - \bar x} \right)^3}}{{\sigma ^3}}\frac{1}{N}$$

where $\bar x$ is the mean, $\sigma$ is the standard deviation, *N* is the number of points, and $x_i$ is the value. The skewness can be positive or negative. A positive skewness has an extended tail to the right of the distribution (median and mean to the right of the most probable value), and a negative skewness has an extended tail to the left of the distribution (median and mean to the left of the most probable value). $Sk = 0$ indicates a perfect fit to the distribution. In general, $|Sk| \,< \,0.5$ is considered small, $\left| {Sk} \right|$ from 0.5–1.0 is considered moderate and $\left| {Sk} \right|\, > \,1.0$ is considered large.

### Principal component analysis

To assess how heart rate and step rate metrics were distributed across the subjects we performed principal component analysis (PCA). Analysis was performed using the PCA function in MATLAB, and for all parameters the mean was set to 0 and standard deviation = 1 using the “Normalize” function. Patients with at least 10 weeks of data were included in the analysis (*N* = 20). Two subjects (PAH4 and 22) were excluded as they had 8 and 7 weeks of data, respectively. Here we report analysis based on 5 metrics: HR(SR = 0):mean, HR(SR > 0):SD, SR(SR > 0):mean, SR(SR > 0):SD, time inactive (fraction of minutes with SR = 0). These parameters were selected to represent heart rate and ambulation metrics and to avoid redundancy. For each parameter we used the average weekly value. The variance for the first two principal components were 48.6% and 30.0%, respectively. For 100 independent runs where we randomly selected different weeks, the mean variance of PC1 and PC2 was 77.5 ± 0.58%.

### Latent Profile Analysis (LPA)

LPA was used to identify the clusters of individuals (i.e. latent profiles) based 8 Fitbit metrics: daily step count, HR(SR = 0), HR(SR = 0)sk, HR(SR > 0), ambulation *P* value, fitness slope, FL6MWD, and usage. LPA was performed through package ‘mclust’ (version 5.4.10) in R (version 4.2.1). The optimal number of clusters was determined based on the maximum Bayesian Information Criterion (BIC) through the function ‘mclustBIC’.

### Derived parameters

From the baseline metrics for step rate and heart rate we obtained the following derived parameters.

#### Usage

The subjects were instructed to wear the device on the non-dominant wrist, for as long as possible, except during exposure to water. The device was considered worn for any given minute if the average heart rate was within the defined range from 20 to the age-determined maximal heart rate. From the complete data set (3.5 × 10^6^ min) there were no HR values less than 20, and only 148 values (4.2 × 10^−7^%) above the age predicted heart rate maximum. To visualize the usage for each individual we created heat maps showing the average usage over each hour of each day between the two clinic visits. We also determined the maximum length of time during the week when the device was not worn, which varied from less than one hour to more than 12 h. The change in weekly usage was determined from a linear least-squares fit to the weekly usage following smoothing using single exponential smoothing with α = 0.3. To assess whether there was a linear relationship between Fitbit parameters we used the Pearson correlation method.

#### Fraction of time inactive

We assessed the fraction time that a subject was inactive each week from the number of minutes with SR = 0 divided by the total number of minutes that the device was worn, based on the criteria described above.

#### Free-living 6MWD

A proxy of the standard 6MWD was obtained by identifying the 6-minute window with the maximum cumulative number of steps during a given week. The step count was converted to a distance from the following relations: step length (m) = 0.413 x height (m) for females and 0.415 x height (m) for males^39^. The free-living 6MWD (FL6MWD) was then obtained from the step length and the total number of steps in the 6 min window. Comparison of step length from different methods is provided in Supplementary Fig. 19.

To compare the FL6MWD to the 6MWD obtained at the clinical visits, we used the *k*-means algorithm (Python 3.8, sklearn.cluster.KMeans). For every week *i*, we determined the difference, $\Delta y_i$ (m), between the FL6MWD and the interpolated clinic value. The average difference over all weeks for an individual was defined as $\overline {\Delta y}$. Using the silhouette method (Python 3.8, sklearn.metrics module), we identified two sub-groups: subjects with FL6MWD close to the interpolated clinic values (small $\overline {\Delta y}$, group 1: “performers”), and those with FL6MWD values below the interpolated clinic values (large $\overline {\Delta y}$, group 2: “underperformers”). An example is provided in Supplementary Fig. 25. The change in weekly FL6MWD with time between the two clinic visits was determined from a linear least-squares fit to the weekly values following smoothing using an exponential smoothing function with α = 0.3 (see Supplementary Fig. 27 for details).

#### Ambulations

Parameters related to intensity, endurance, and frequency of ambulations were obtained from analysis of the weekly step rate data. An ambulation event was defined by a sustained step rate of ≥60 SPM for at least 2 min. For a healthy individual, a step rate of 60–79 SPM is considered slow walking^23^. The ambulation intensity was defined as the standard deviation of a lognormal fit to the weekly step rate histogram. We defined endurance as the 1/e value of an exponential fit (starting at 2 min) to a histogram of the ambulation duration. Ambulation frequency was defined as the total number of ambulations during the week. Finally, we defined a characteristic ambulation parameter *P* for each subject, based on the product of the average values of intensity, endurance, frequency.

#### Fitness

A proxy of fitness was obtained using an approach similar to the Physical Working Capacity PWC170 protocol^40^. In the submaximal PWC170 test, an individual is asked to spin on a stationary bike at two or more power outputs that maintain the heart rate within a defined range. The power output at an extrapolated heart rate of 170 bpm is then considered a proxy of VO_2,max_. We used a similar approach to define a metric of fitness, taking the step rate as a proxy for level of effort, and hence assuming that that higher step count corresponds to higher power output. The mean and standard deviation of the step count per minute in 20 SPM bins was plotted versus the mean and standard deviation of the heart rate in that bin for each week. We then determined the slope (HR/SPM) and intercept (HR at SR = 0) from the weekly plots. To compare subjects, we plotted the mean and standard deviation of step rate and heart rate in each bin for each subject averaged over all weeks. We then identified the mean and standard deviation for all subjects within each bin.

#### Physical health state

An estimate of the physical health state of each individual was determined from FL6MWD/H6MWD, where H6MWD is the value of the 6MWD predicted for an equivalent healthy individual. Various studies have shown that 6MWD values for healthy individuals are dependent on age, gender and BMI (height and weight)^33,41^. H6MWD (m) was calculated from^41^:

2 $$H6MWT = 890.46 - (6.11xage) + (0.0345xage^2) + (48.87xgender) - (4.87xBMI)$$

where age is in years, gender = 0 (female) and 1 (male), and BMI is in units of kg m^−2^. This empirical relation was derived from measurements of 617 subjects (52% female) aged 29–79 with BMI in the range of 18 kg m^−2^–40 kg m^−2^ who completed two 6MWTs. The fit to the data captured 46% of the variance. Other studies of healthy individuals were excluded because they had a narrower age range^42,43^ or a narrower range of BMI^44^.

### Comparison to clinical data

To identify clinical parameters that were statistically significant between sub-groups determined from Fitbit-derived metrics we used a Mann-Whitney test. If a subject did not have a value for a specific clinical parameter, then that parameter was excluded from the analysis. Comparisons between groups were only made if there were at least 5 subjects in each group. No other corrections were made.

### Reporting summary

Further information on research design is available in the Nature Research Reporting Summary linked to this article.

## Supplementary information

Supplementary Material

Reporting Summary Checklist

## Data Availability

The data that support the findings of this prospective, observational study are not openly available but are available from the authors upon reasonable request. The data presented here are available within the article or supplementary materials.

## Code availability

Scripts for data analysis are available at https://github.com/peterxu1991/PAH-Fitbit-Paper.
