# Supplementary material for: Evaluation of physical health status beyond daily step count using a wearable activity sensor
Source: NPJ Digit Med. 2022 Nov 9;5:164. doi: 10.1038/s41746-022-00696-5 (PMC9646807; doi:10.1038/s41746-022-00696-5)
Supplement: Supplementary file 1 — Supplementary Material [file 41746_2022_696_MOESM1_ESM.pdf]

SUPPLEMENTARY INFORMATION

Zheng Xu,[1,2] Nicole Zahradka,[1,2] Seyvonne Ip,[1,2] Amir Koneshloo,[1,2] Ryan T. Roemmich,[3,4] Sameep Sehgal,[5] Kristin B. Highland,[5] and Peter C. Searson[1,2,3,6,7]

[1] Measurement Corps, In Health, Johns Hopkins University School of Medicine
[2] Institute of Nanobiotechnology, Johns Hopkins University
[3] Center for Movement Studies, Kennedy Krieger Institute
[4] Department of Physical Medicine and Rehabilitation, Johns Hopkins University School of Medicine
[5] Respiratory Institute, Cleveland Clinic, Cleveland, OH
[6] Department of Biomedical Engineering, Johns Hopkins University
[7] Department of Materials Science & Engineering, Johns Hopkins University

Supplementary Tables

Table 1. Summary of clinical parameters
Table 2. Summary of clinically-relevant parameters based on Fitbit-derived metrics.
Table 3. Bayesian Information Criteria (BIC) values for the multivariate ellipsoidal equal shape (VEV) model.
Table 4. Average values of Fitbit metrics within each group identified from LPA analysis.

Supplementary Figures

Fig. 1. Mean daily step count.
Fig. 2. Clinical parameters with statistical significance for subjects with average daily step counts greater than or less than 5,000.
Fig. 3. Sensitivity analysis of thresholds for Fitbit metrics in comparison to clinical parameters. Only clinical parameters with p-values < 0.05 are shown.
Fig. 4. Number of statistically significant clinical parameters for different threshold values of Fitbit metrics.
Fig. 5. Average resting heart rate HR(SR=0).
Fig. 6. Skewness of HR(SR=0).
Fig. 7. Examples of the distribution of HR(SR=0).
Fig. 8. Clinical parameters with statistical significance for subjects with mean HR(SR=0) (RHR) > 82 BPM compared to HR(SR=0) < 82 BPM.
Fig. 9. Clinical parameters with statistical significance for subjects with skewness of HR(SR=0) > 1 compared to skewness of HR(SR=0) < 1.
Fig. 10. Values for mean HR(SR>0).
Fig. 11. Clinical parameters with statistical significance for subjects with mean HR(SR>0) > 95 BPM (10/22) compared to mean HR(SR>0) < 95 BPM (12/22).
Fig. 12. PCA plots highlighting patient subgroups.
Fig. 13. Clinical parameters with statistical significance for subjects with fitness slope > 0.15 (11/22) compared to those with fitness slope < 0.15 (11/22).

Fig. 14. Clinical parameters with statistical significance for subjects with ambulation product P (= frequency × duration × intensity) > 1000 (12/22) compared to those with P < 1000 (10/22).
Fig. 15. Heat maps showing device usage for 22 subjects.
Fig. 16. The maximum off-time for each week for all 22 subjects. Each point represents the usage and corresponding maximum-off time for each week.
Fig. 17. Average weekly device usage for all subjects. Blue line – mean; green lines – upper and lower quartiles.
Fig. 18. Clinical parameters with statistical significance for subjects with usage > 0.94 (7/22) compared to those with usage < 0.94 (15/22).
Fig. 19. Examples of smoothing of weekly usage data.
Fig. 20. Change in average weekly device usage over time.
Fig. 21. Conversion of step rate (SPM) to step length.
Fig. 22. Clinic 6MWD and FL6MWD for all subjects.
Fig. 23. Clinical parameters with statistical significance for subjects with average FL6MWD > 320 m (14/22) compared to subjects with values < 320 m (8/22).
Fig. 24. The mean difference $\overline{\Delta y}$ between the interpolated clinic values (6MWD) and the FL6MWD was determined from the average of the weekly values Δy_i for each subject.
Fig. 25. Demographic and clinical parameters with statistical significance between group 1 and group 2.
Fig. 26. Example of smoothing of FL6MWD.
Fig. 27. Example of smoothing of FL6MWD.
Fig. 28. Latent profile analysis using 8 Fitbit metrics.
Fig. 29. Results from PCA analysis showing location of the three groups identified from LPA analysis.

Supplementary References

Supplementary Table 1. Summary of clinical parameters

Clinic Measures	Description	Range	Clinical Meaning of Score/Value
Hemoglobin	protein		normal levels: males: 14 - 18 g/dL; females: 12 - 16 g/dL<br>low values (anemia): blood loss, nutritional deficiency, kidney failure, …<br>high values: lung disease, …
Albumin	protein		normal range: 3.4 to 5.4 g/dL (34 to 54 g/L)<br>low values: kidney disease, liver disease, Crohn's diseases, celiac disease, …<br>high values: dehydration, high protein diet, …
NT-proBNP	Protein:<br>N-terminal prohormone of brain natriuretic peptide		normal range:<br>< 125 pg/mL (<74 y/o)<br>< 450 pg/mL (>75 y/o)<br>High values associated with congestive heart failure and coronary artery disease<br>heart function could be unstable:<br>> 450 pg/mL (< 50 y/o)<br>> 900 pg/mL (> 50 y/o)
EQ-5D Self-Care	EuroQol 5 Dimension (EQ-5D) 5-level (5L)	1 to 5	1 = I have no problem washing or dressing myself; 5 = I am unable to wash or dress myself
EQ-5D Mobility	EQ-5D-5L	1 to 5	1 = I have no problem in walking about; 5 = I am unable to walk about
EQ-5D Usual Activity	EQ-5D-5L	1 to 5	1 = I have no problem doing my usual activities; 5 = I am unable to do my usual activities
EQ-5D Pain / Discomfort	EQ-5D-5L	1 to 5	1 = I have no pain or discomfort; 5 = I have extreme pain or discomfort
EQ-5D Anxiety / Depression	EQ-5D-5L	1 to 5	1 = I am not anxious or depressed; 5 = I am extremely anxious or depressed
EQ VAS Score	EuroQol Visual Analog Scale (EQ VAS): self-assessment of an individual's health	0 to 100	100 = best health you can imagine<br>0 = worst health you can imagine
EQ-5D Index	index derived from weighted scores derived from EQ-5D-5L	0 to 1	1 = full health; 0 = death
VAS Score	patient self-assessment of the severity of PAH over the past 28 days	0 to 100	0 = lowest severity<br>100 = highest severity
Resting Heart Rate (RHR)	value of heart rate while sitting before 6MWT		typically 60 to 100 BPM
Borg Dyspnea Score	self-assessment of breathing difficulty during submaximal exercise (e.g. 6MWT)	0 to 10	modified Borg dyspnea score<br>0 = no difficulty breathing<br>10 = breathing difficulty is maximal
Six Minute Walk Distance (6MWD)	distance an individual is able to walk in six minutes on a hard, flat surface		poor outcomes for < 320 m<br>therapeutic goal: > 400 m
Peak HR	maximum HR during 6MWT		peak HR typically 70% of max HR or above<br>maximum HR (BPM) = 220 – age (years)
Cough	a sudden, forceful hacking sound to release air and clear an irritation in the throat or airway	1 or 2	1 = present; 2 = absent
Pedal Edema	fluid in feet and lower legs	1 or 2	1 = present; 2 = absent
Palpitations	a sensation that the heart is racing, pounding, fluttering, or skipping a beat; usually harmless	1 or 2	1 = present; 2 = absent

Angina	type of chest pain caused by reduced blood flow to the heart	1 or 2	1 = present; 2 = absent
Syncope	fainting, or a sudden temporary loss of consciousness; can occur due to a sudden drop in blood pressure, a drop in heart rate, or changes in the amount of blood in areas of your body	1 or 2	1 = present; 2 = absent
Chest Pain	discomfort in the chest including a dull ache, a crushing or burning feeling, a sharp stabbing pain, and pain that radiates to the neck or shoulder.	1 or 2	1= present; 2 = absent
WHO FC (physician)	WHO Functional Class (FC) assessment for pulmonary hypertension evaluated by the physician	1 to 4	**Class I**: Patients with pulmonary hypertension but without resulting limitation of physical activity. Ordinary physical activity does not cause undue dyspnea or fatigue, chest pain or near syncope. ("Doing great")<br>**Class II**: Patients with pulmonary hypertension resulting in a slight limitation of physical activity. They are comfortable at rest. Ordinary physical activity causes undue dyspnea or fatigue, chest pain or near syncope. ("Acceptable")<br>**Class III**: Patients with pulmonary hypertension resulting in marked limitation of physical activity. They are comfortable at rest. Less than ordinary activity causes undue dyspnea or fatigue, chest pain or near syncope. ("Caution")<br>**Class IV**: Patients with pulmonary hypertension with inability to carry out any physical activity without symptoms. These patients manifest signs of right heart failure. Dyspnea and/or fatigue may even be present at rest. Discomfort is increased by any physical activity. ("Emergency")
WHO FC (patient)	WHO Functional Class (FC) assessment for pulmonary hypertension evaluated by the patient		
RV Function	Right ventricular dysfunction score calculated from the summation of points associated with four parameters (TAPSE < 16 mm, 1 point; S′ < 10 cm/s, 1 point; RVFAC < 35%, 1 point; and RV-MPI > 0.4, 1 point) using the cut-off value recommended by ASE guidelines	0 to 4	higher score indicates worse RV function
Right Ventricular Systolic Pressure (RVSP)	right ventricular systolic pressure		normal range: 35 to 36 mmHg
Systolic Murmur	heart murmur (sounds — such as whooshing or swishing — due to turbulent blood in or near the heart) when the heart is emptying	1 or 2	1 = present; 2 = absent

Supplementary Table 2. Summary of clinically-relevant parameters based on Fitbit-derived metrics. p-values determined from a Mann-Whitney test based on the indicated threshold value.

Fitbit Metric	Threshold	Clinical Parameter	Visit	p value
steps/day	<5,000	lower 6MWD	1	0.0288
		lower right ventricular systolic pressure (RVSP)	1	0.0148
		lower hemoglobin	2	0.0126
		more severe disease (higher physician-assessed WHO FC)	1	0.0233
		experienced more persistent coughing (lower Coughing score)	1	0.0273
		experienced more pedal edema (lower Pedal Edema score)	2	0.0315
HR(SR=0)	<82 BPM	lower RHR	1	0.0006
		lower RHR	2	0.0011
		lower peak HR	2	0.0183
		lower NT-proBNP levels	1	0.0397
		less able to perform usual activities (higher EQ-5D Usual Activity score)	1	0.0356
		experienced more pain/discomfort (higher EQ-5D pain/discomfort score)	1	0.0258
		experienced more pedal edema (lower Pedal Edema score)	1	0.0243
		experienced more palpitations (lower Palpitation score)	1	0.0378
HR(SR=0) skewness	<1	higher RHR	1	0.0233
		higher RHR	2	0.0376
		experienced less pain/discomfort (lower EQ-5D pain/discomfort score)	1	0.0049
		better health (higher EQ-5D Index)	1	0.0321
HR(SR>0)	<95 BPM	lower RHR	1	0.0041
		lower RHR	2	0.0012
		lower albumin levels	1	0.0221
		experienced more palpitations (lower Palpitation score)	1	0.0118
Fitness slope	<0.15 BPM/SPM	higher NT-proBNP levels	1	0.0024
		higher NT-proBNP levels	2	0.0176
		higher RV pressure (poorer RV function)	1	0.0071
Ambulation product	P<1000	lower 6MWD	1	0.0228
		lower 6MWD	2	0.0348
		lower peak heart rate	1	0.0345
		lower right ventricular systolic pressure (RVSP)	1	0.0263
		lower hemoglobin levels	2	0.0148
		experienced more pedal edema (lower Pedal Edema score)	1	0.0430
		experienced more persistent coughing (lower Coughing score)	2	0.0292
Device usage	< 0.94	more severe PAH (higher EQ VAS score)	1	0.0311
		worse pulmonary health (higher physician-assessed WHO FC)	1	0.0264
		experienced more pedal edema (lower Pedal Edema score)	2	0.0260
		more difficulty breathing (higher Modified Borg Dyspnea score)	2	0.0437
FL6MWD	<320m	lower 6MWD	1	0.0127
		lower 6MWD	2	0.0127
		experienced more pedal edema (lower Pedal Edema score)	2	0.0315
		worse pulmonary health (higher physician-assessed WHO FC)	1	0.0053
		experienced more persistent coughing (Coughing score)	1	0.0273
		lower hemoglobin	2	0.0107

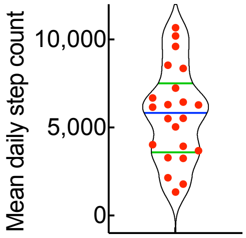

Supplementary Figure 1. Mean daily step count. The average daily step count for each subject ranged from 1,338 - 10,679 steps per day. The mean value for all subjects was 5,729 steps per day. Blue bar – mean; green bars – upper and lower quartiles.

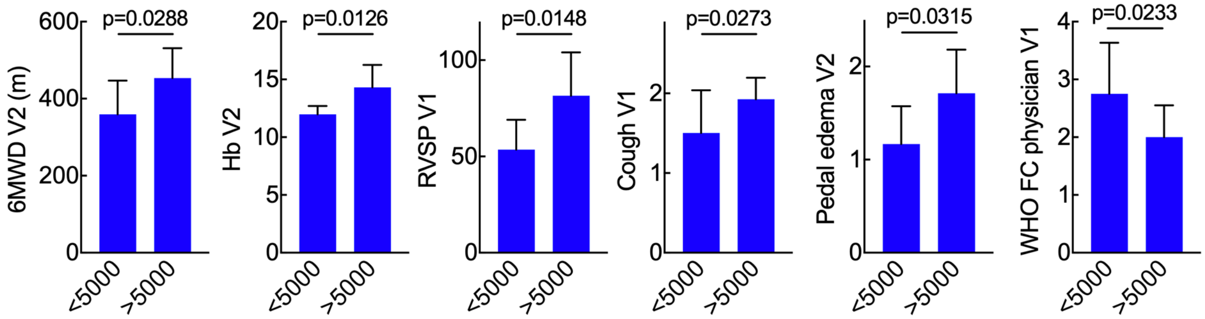

Supplementary Figure 2. Clinical parameters with statistical significance (Mann-Whitney test) for subjects with average daily step counts greater than or less than 5,000. Bars represent mean ± SD.

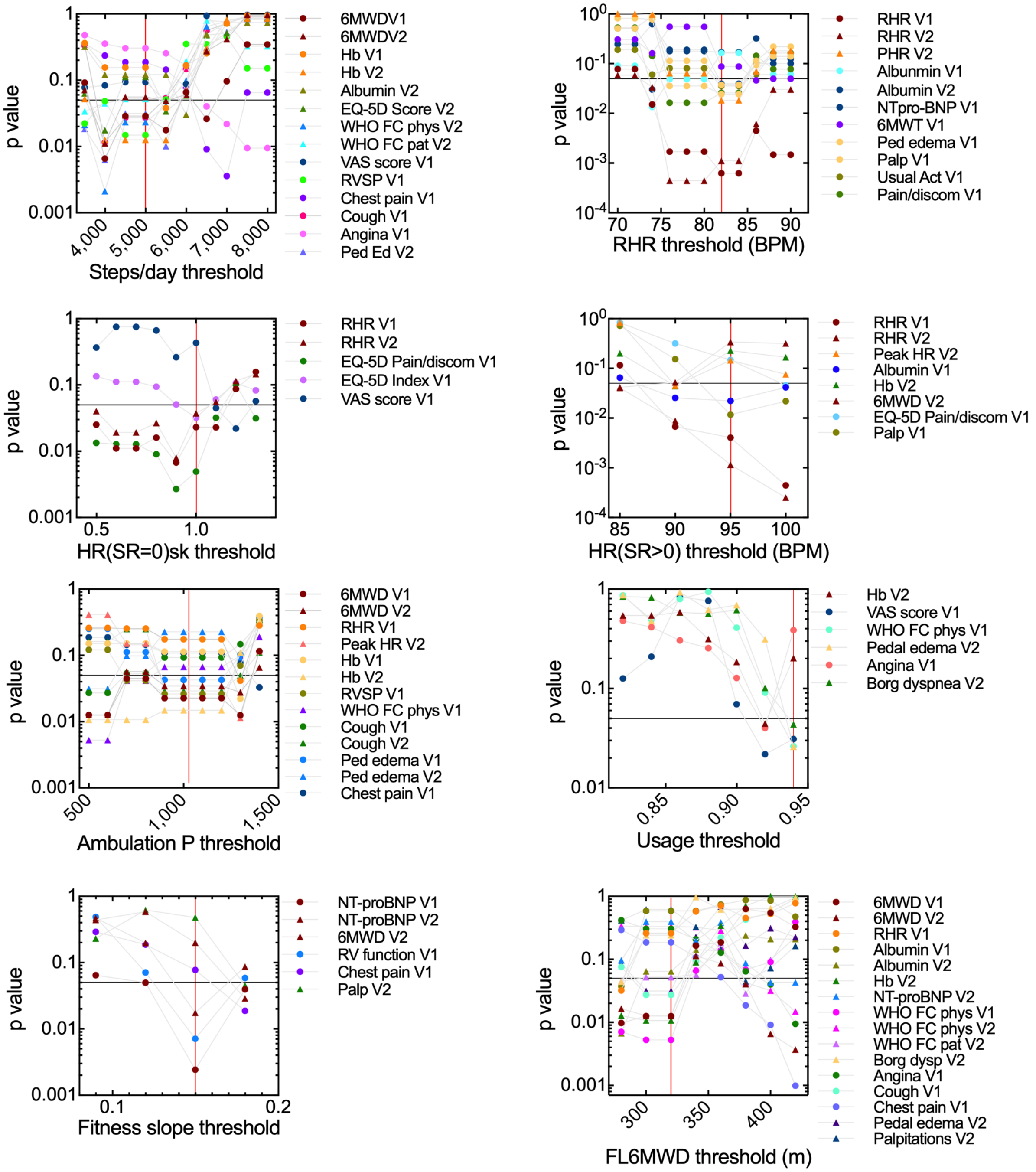

Supplementary Figure 3. Sensitivity analysis of thresholds for Fitbit metrics in comparison to clinical parameters. For 8 Fitbit metrics we calculated the statistical significance (Mann-Whitney test) between groups (for n ≥ 5 in each group) for all clinical parameters. Only clinical parameters with p-values < 0.05 are shown. p-values determined from a Mann-Whitney test.

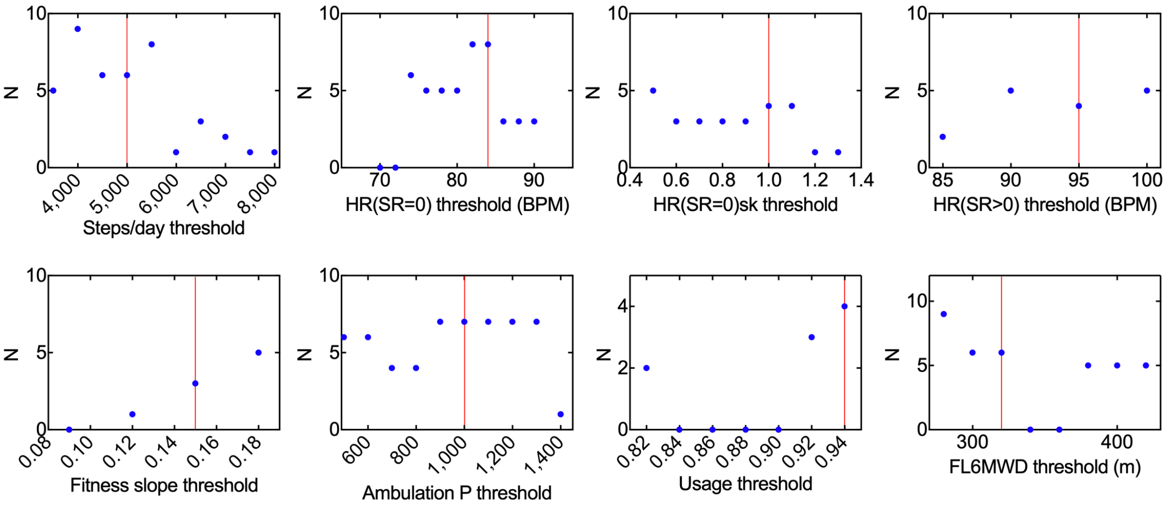

Supplementary Figure 4. Number of statistically significant clinical parameters for different threshold values of Fitbit metrics. The red line indicates the threshold value used for analysis. Based on p-values < 0.05 using a Mann-Whitney test.

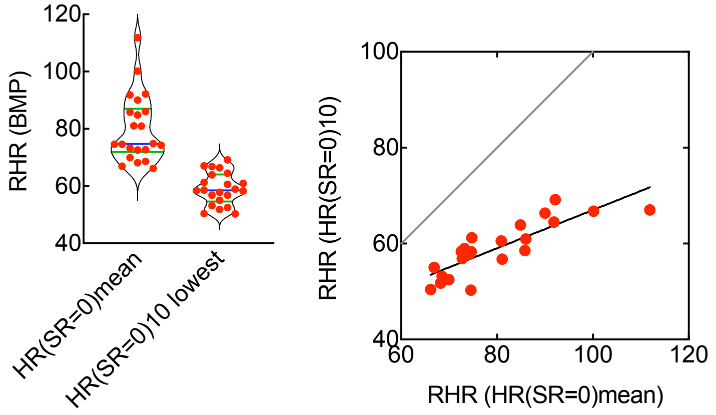

Supplementary Figure 5. Average resting heart rate HR(SR=0). (A) Violin plots of resting heart rate: mean value of HR(SR=0) and the mean of the lowest 10 values of HR(SR=0). Mean HR(SR=0) = 80.1 BPM, Mean HR(SR=0)lowest 10 = 59.1 BPM. Blue bar – mean; green bars – upper and lower quartiles. (B) Correlation plot between mean HR(SR=0) and the average of the lowest 10 values of HR(SR=0) for each week. Pearson correlation coefficient = 0.851.

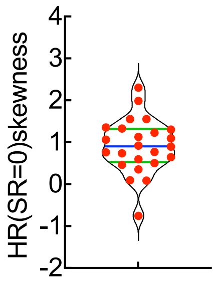

Supplementary Figure 6. Values of the mean skewness of resting heart rate (HR(SR=0)) for each subject. Mean = 0.93. Blue bar – mean; green bars – upper and lower quartiles.

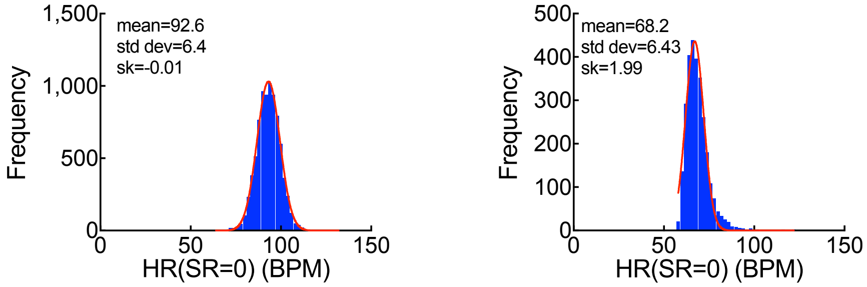

Supplementary Figure 7. Examples of the distribution of resting heart rate over one week (HR(SR=0)). (A) PAH 14 at week 15, skewness = -0.01. (B) PAH 27 at week 1, skewness = 1.99. For PAH 27, the mean is higher than the most probable value and there is an increased tail at high HR.

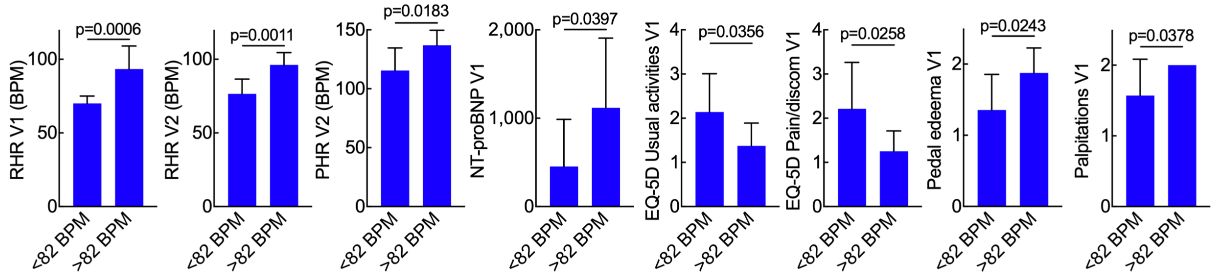

Supplementary Figure 8. Comparison of mean resting heart rate while inactive (HR(SR=0) (Fitbit) for subgroups with statistically significant differences in clinical parameters. Clinical parameters with statistical significance (Mann-Whitney test) for subjects with mean HR(SR=0) > 82 BPM (8/22) compared to HR(SR=0) < 82 BPM (14/22). Bars represent mean ± SD.

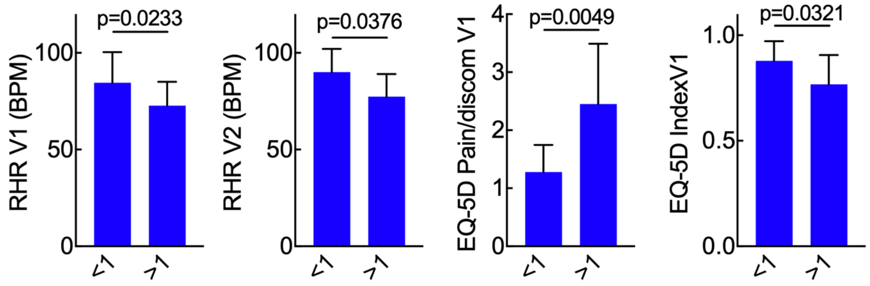

Supplementary Figure 9. Comparison of the skewness of resting heart rate (HR(SR=0)sk)) (Fitbit) for subgroups with statistically significant differences in clinical parameters. Clinical parameters with statistical significance (Mann-Whitney test) for subjects with skewness of HR(SR=0) > 1 (11/22) compared to skewness of HR(SR=0) < 1 (11/22). Bars represent mean ± SD.

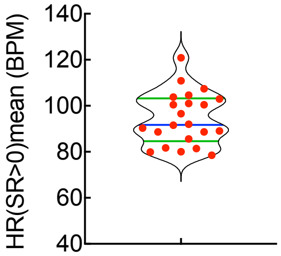

Supplementary Figure 10. Values for mean heart rate while active (HR(SR>0)) for each subject. Mean = 94.4 BPM. Blue bar – mean; green bars – upper and lower quartiles.

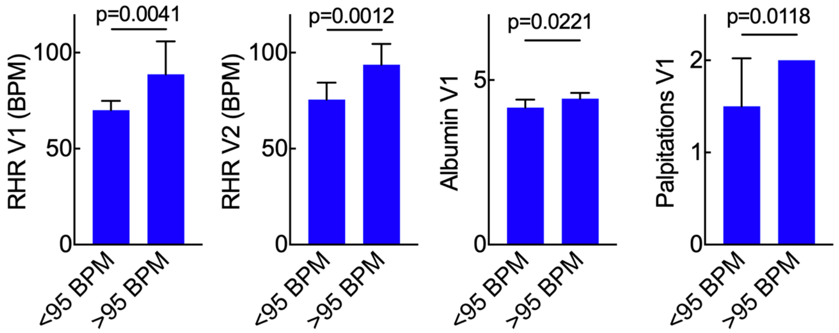

Supplementary Figure 11. Comparison of the mean heart rate while active (SR>0) (Fitbit) for subgroups with statistically significant differences in clinical parameters. Clinical parameters with statistical significance (Mann-Whitney test) for subjects with mean HR(SR>0) > 95 BPM (10/22) compared to mean HR(SR>0) < 95 BPM (12/22). Bars represent mean ± SD.

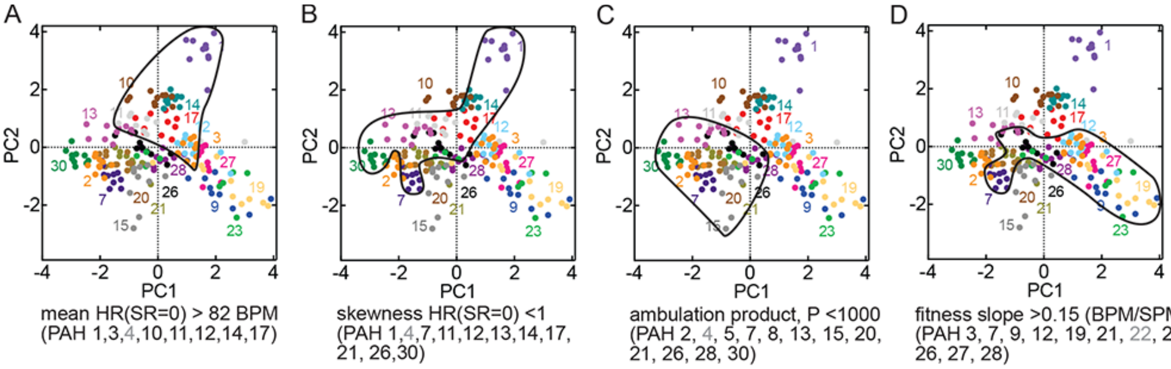

Supplementary Figure 12. PCA plots highlighting patient subgroups. (A) Subjects with mean HR at SR=0 > 82 BPM. (B) Subjects with skewness of the HR at SR=0 of <1. (C) Subjects with ambulation product, *P* (see text for details) < 1000. (D) Subjects with a fitness plot slope > 0.15. Note that subjects PAH4 and PAH22 had 8 and 7 weeks of data, respectively, and hence were excluded from analysis. Numbers represent subject IDs. Each point represents one week of data.

Several subgroups of patients defined by Fitbit parameters were clustered in the PCA plots. The weekly data for subjects with mean HR at SR=0 > 82 BPM are located in the upper two quadrants. Subjects with skewness of HR at SR=0 < 1 are located along the negative x-axis and positive y-axis. Subjects with ambulation product *P* < 1000 are located primarily in the fourth quadrant. Subjects with a fitness slope > 0.15 tend to be located in the fourth quadrant or close to the origin. Other subgroups were distributed across the PCA plot with no obvious clustering. These include:

the performers and underperformers in the FL6MWT, mean HR at SR>0 +/- 95 BPM, and parameters associated with usage.

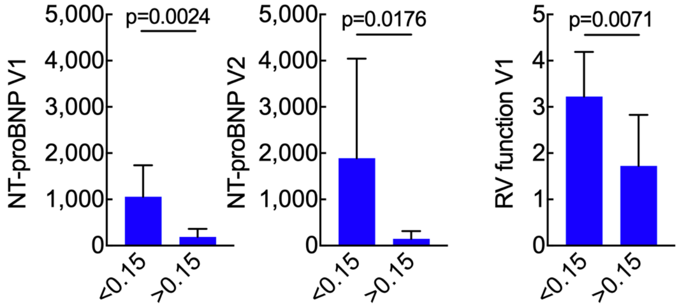

Supplementary Figure 13. Comparison of the fitness slope (Fitbit) for subgroups with statistically significant differences in clinical parameters. Clinical parameters with statistical significance (Mann-Whitney test) for subjects with fitness slope > 0.15 (11/22) compared to those with fitness slope < 0.15 (11/22). Bars represent mean ± SD.

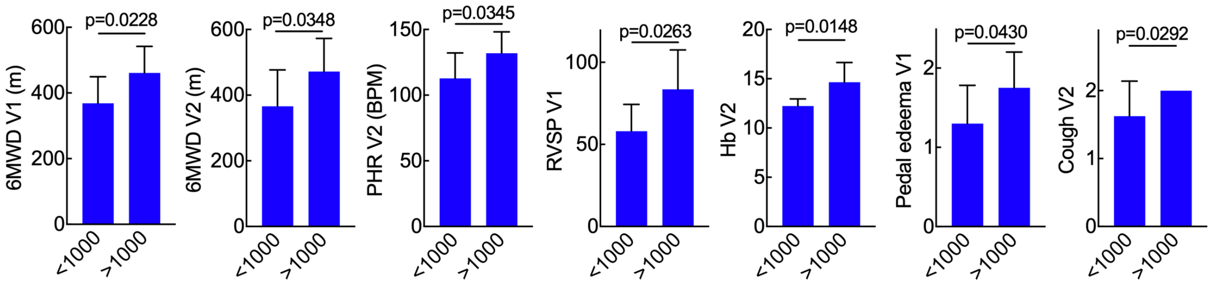

Supplementary Figure 14. Comparison of ambulation product *P* (Fitbit) for subgroups with statistically significant differences in clinical parameters. Clinical parameters with statistical significance (Mann-Whitney test) for subjects with ambulation product *P* (= frequency × duration × intensity) > 1000 (12/22) compared to those with *P* < 1000 (10/22). Bars represent mean ± SD.

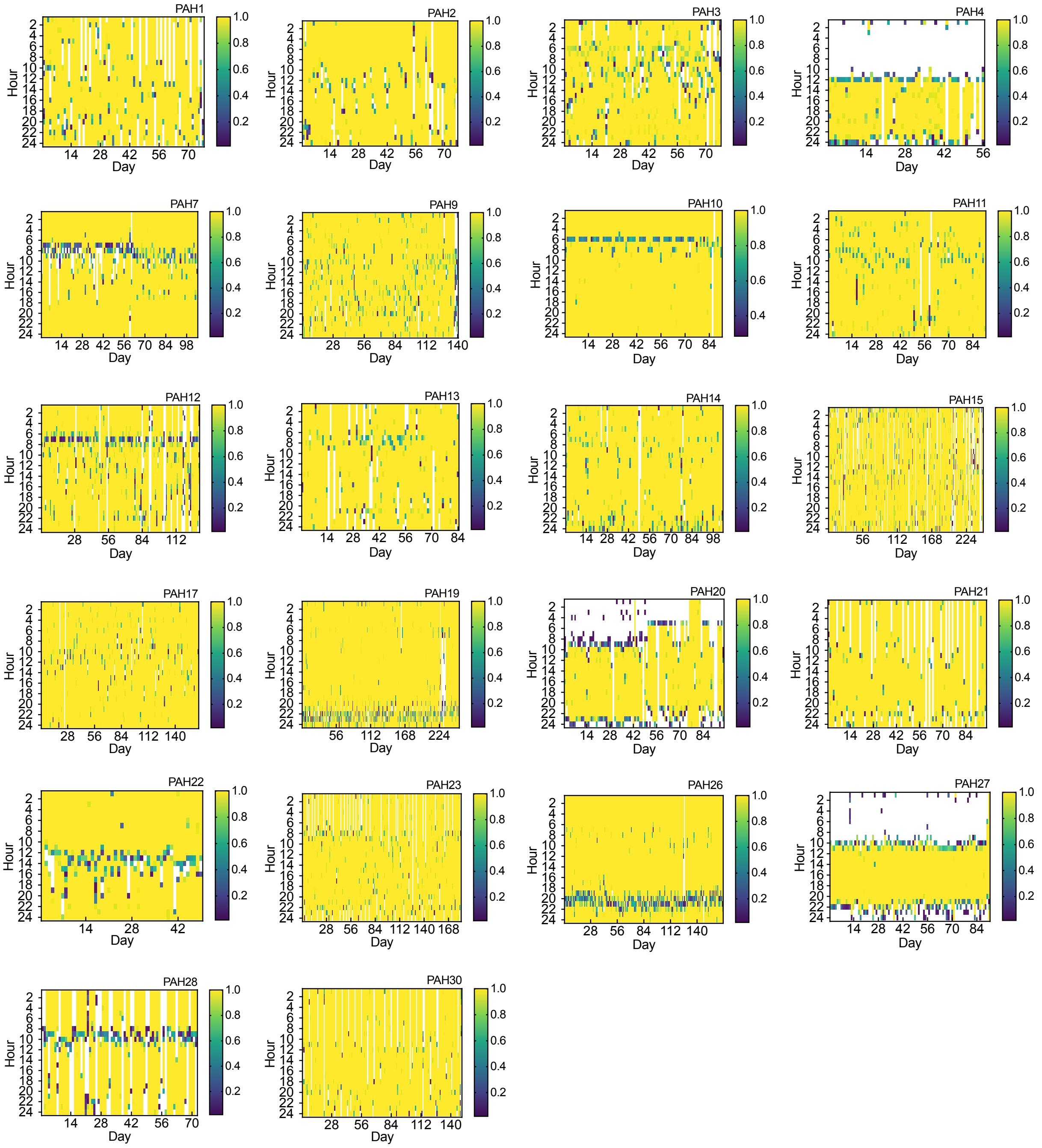

Supplementary Figure 15. Heat maps showing device usage for 22 subjects. Yellow cells indicate that the device was worn for the full hour. White cells indicate that the device was not worn (no HR recorded) for the full hour.

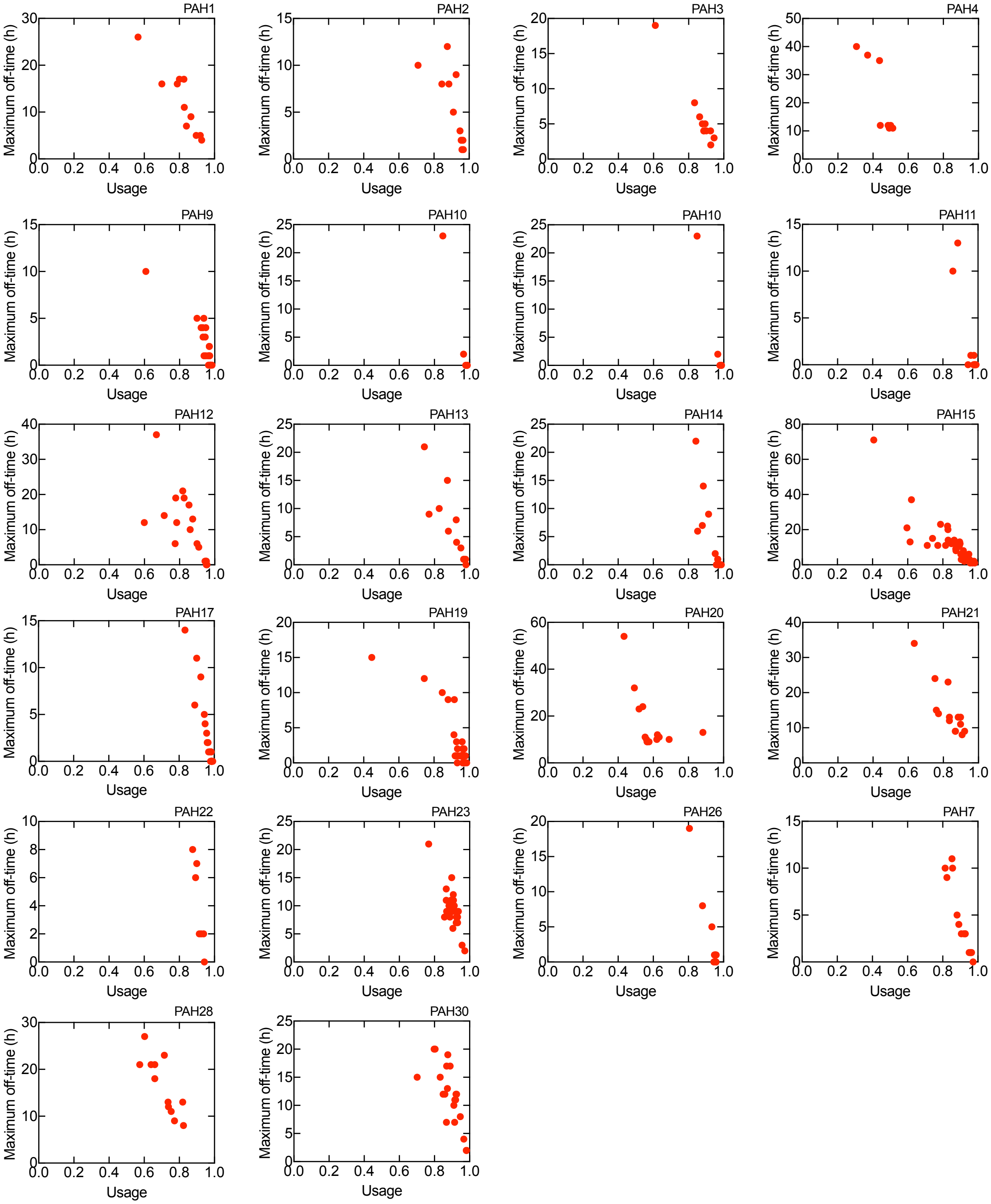

Supplementary Figure 16. The maximum off-time for each week for all 22 subjects. Each point represents the usage and corresponding maximum-off time for each week.

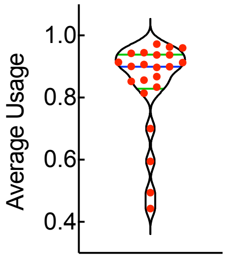

Supplementary Figure 17. Values of average weekly device usage for all subjects. Blue bar – mean; green bars – upper and lower quartiles.

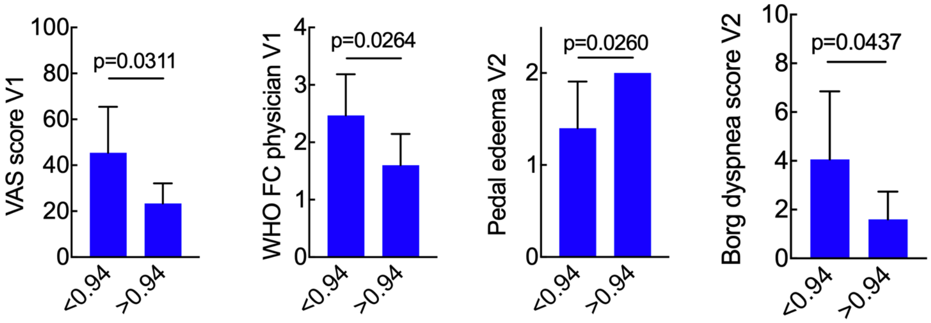

Supplementary Figure 18. Comparison of ambulation product *P* (Fitbit) for subgroups with statistically significant differences in clinical parameters. Clinical parameters with statistical significance (Mann-Whitney test) for subjects with usage > 0.94 (7/22) compared to those with usage < 0.94 (15/22). A usage of 0.94 corresponds to 75th percentile. Bars represent mean ± SD.

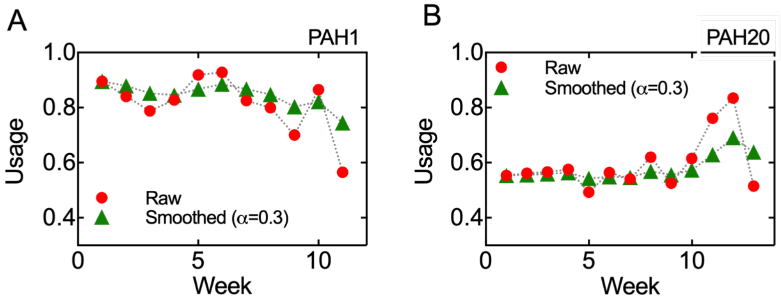

Supplementary Figure 19. Examples of smoothing of weekly usage data. (Circles) raw data, (triangles) smoothed data. The change in weekly usage was determined from a linear least squares fit to the weekly usage values for each subject. Prior to fitting, we performed single exponential smoothing to reduce the influence of individual data points:

$$S_i = \alpha x_i + (1-\alpha)S_{i-1} \quad (i > 1) \qquad (1)$$

$$S_i = x_1 \quad (i = 1) \qquad (2)$$

where S_i is the smoothed value at week i, x_i is the value of usage in week i, and α is the smoothing parameter (0 - 1). Here we used $\alpha = 0.3$. Note that $S_1 = x_1$, i.e. the first value corresponds to the value of usage in week 1.

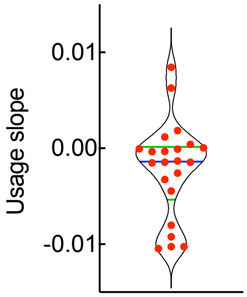

Supplementary Figure 20. Change in weekly average device usage over time. The slope (change in usage per week) represents a linear least squares fit of usage versus time following smoothing (see above). Five subjects (PAH1, 3, 4, 12, 21) showed an average decrease in usage more negative than -0.006 (0.6% per week). Blue bar – mean; green bars – upper and lower quartiles. Changes in weekly usage were in the range from 0.011 (+1.1%/week) to -0.022 (-2.2% per week). Subjects with a decrease in weekly usage < -0.006 (0.6%/week) had higher resting heart rate at visit 1 ($p = 0.03$) and visit 2 ($p = 0.0004$), were more able to perform usual activities (lower EQ-5D Usual Activity scores) at visit 1 ($p = 0.04$), had higher albumin levels at visit 1 ($p = 0.009$), were less able to look after themselves (higher EQ-5D Self-Care score) at visit 2 ($p = 0.04$), experienced fainting (Syncope score) at visit 2 ($p = 0.04$), and had lower RVSP at visit 2 ($p = 0.04$). Five subjects (PAH 1, 3, 4, 12, 21) showed an average decrease in usage more negative than -0.006 (0.6% per week), and all had an average usage < 0.94. Although there was no statistically significant correlation between usage and sex or age, these data can be useful in planning studies with different demographics. p-values obtained from a Mann-Whitney test.

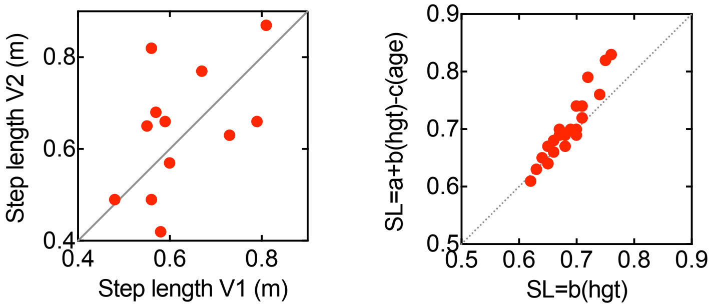

Supplementary Figure 21. Step length. (A) step length obtained from 6MWD performed at clinic visit 1 (V1) and clinic visit 2 (V2). 12/22 subjects had values for both clinic visits that were < 1 m. Pearson correlation coefficient = 0.51. (B) Comparison of predicted step length (SL) of 22 PAH subjects based on height (Equations (3) and (4)) or height and age (Equation (5) and (6)). Pearson correlation coefficient = 0.956.

To convert step rate or step count to distance requires an estimate of step length (stride length/2). From the clinic data for the 6MWD we had both distance and number of steps for 14/22 subjects at both clinic visits. Two subjects were excluded since the number of recorded steps was low, resulting in step lengths > 1 m. Comparison of the step length between visit 1 and visit 2 was surprisingly poor (see Figure) implying that the number of steps recorded during the 6MWT may be unreliable.

Step length for healthy adults is typically in the range from 0.6 – 0.8 m, but is dependent on height and age. A common pair of equations to that are widely used to predict step length based on height is:

$$\text{step length (m)} = 0.413 \times \text{height (m)} \quad \text{(females)} \qquad (3)$$
$$\text{step length (m)} = 0.415 \times \text{height (m)} \quad \text{(males)} \qquad (4)$$

A study of 118 females and 121 males from 19 - 90 years old reported the following pair of equations:[1]

$$\text{step length (m)} = 0.001 + 0.448 \times \text{height (m)} - 0.001 \times \text{age (yo)} \quad \text{(females)} \qquad (5)$$
$$\text{step length (m)} = 0.181 + 0.352 \times \text{height (m)} - 0.001 \times \text{age (yo)} \quad \text{(males)} \qquad (6)$$

Comparison of the two methods for the 22 subjects in this study yield similar values (see Figure), with step length typically in the range from 0.6 - 0.75 m. There are three subjects (PAH 20, 23, 30) that are considerably above the fitted line (all male subjects). In all subsequent analysis we used Equations (3) and (4).

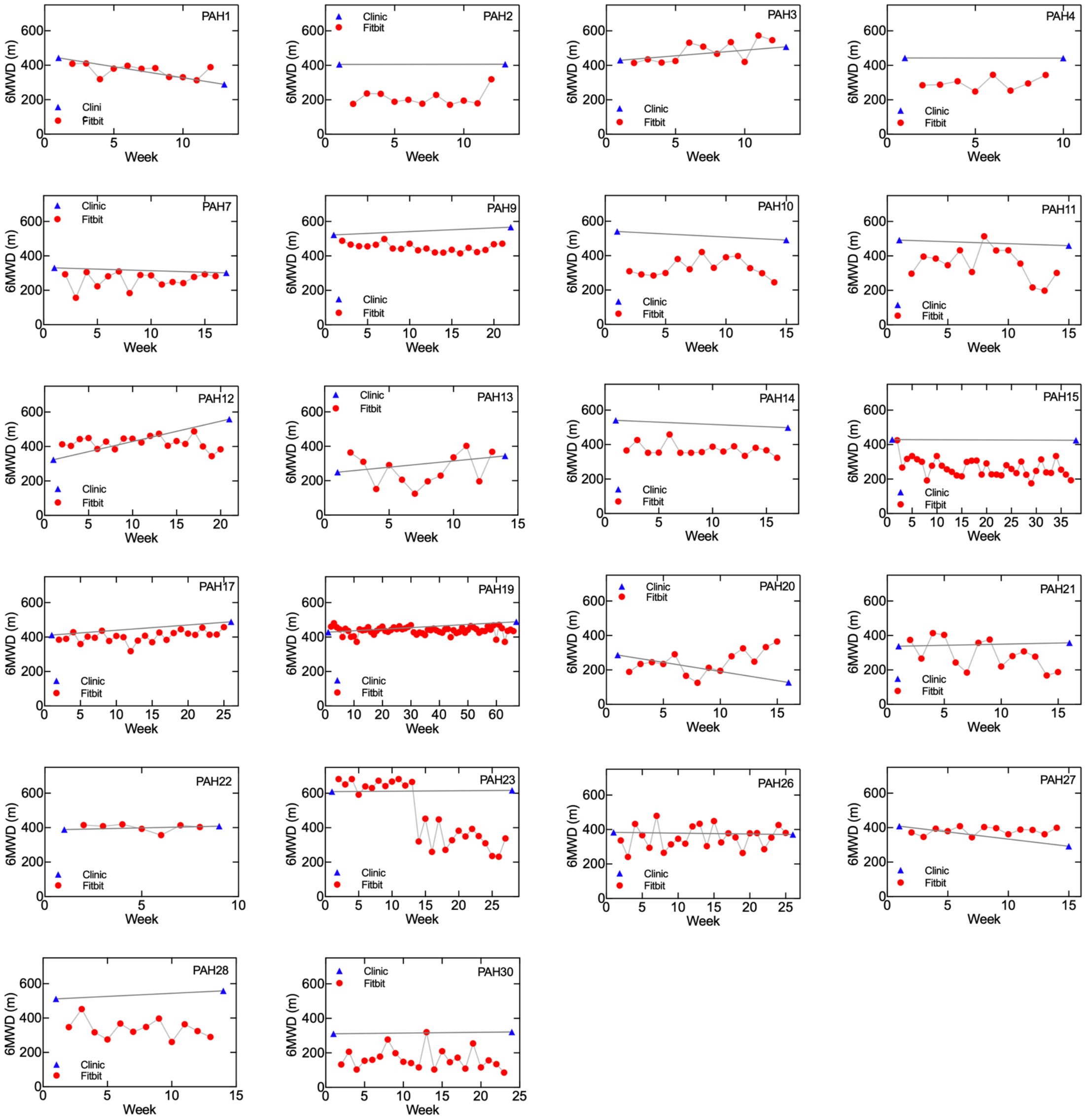

Supplementary Figure 22. Clinic 6MWD and FL6MWD for all subjects. (Triangles) clinic values, (circles) FL6MWD. The grey line shows the linear interpolation of the clinic values. FL6MWD was determined from the continuous block of time with the maximum step count and converted to distance using the predicted step length (Equations (3) and (4)).

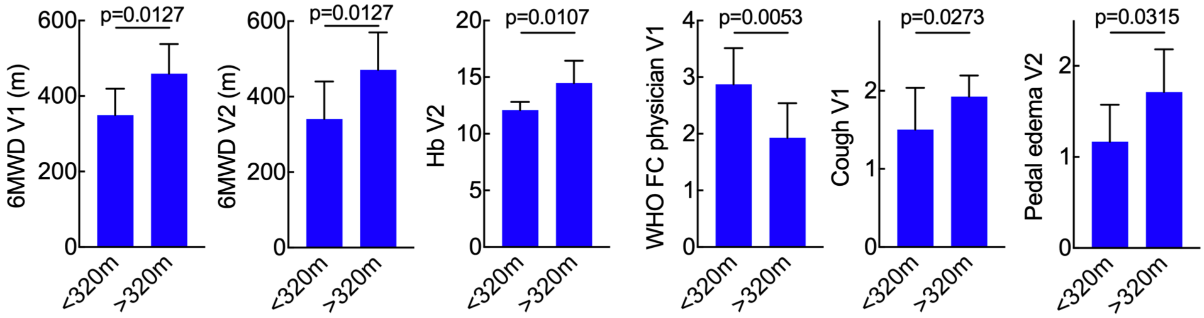

Supplementary Figure 23. Comparison of free living 6 minute walk distance (FL6MWD) (Fitbit) for subgroups with statistically significant differences in clinical parameters. Clinical parameters with statistical significance (Mann-Whitney test) for subjects with average FL6MWD > 320 m (14/22) compared to subjects with values < 320 m (8/22). Subjects with FL6MWD >320 m: PAH 1, 3, 9, 10, 11, 12, 14, 17, 19, 22, 23, 26, 27, 28. Bars represent mean ± SD.

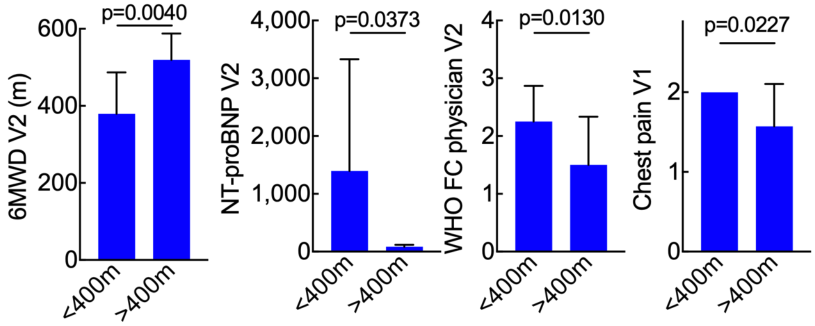

Supplementary Figure 24. Comparison of free living 6 minute walk distance (FL6MWD) (Fitbit) for subgroups with statistically significant differences in clinical parameters. Clinical parameters with statistical significance (Mann-Whitney test) for subjects with average FL6MWD > 400 m (12/22) compared to subjects with values < 400 m (10/22). Subjects with FL6MWD > 400 m: PAH 1, 3, 5, 9, 10, 13, 14, 15, 16, 17, 19, 20. Bars represent mean ± SD.

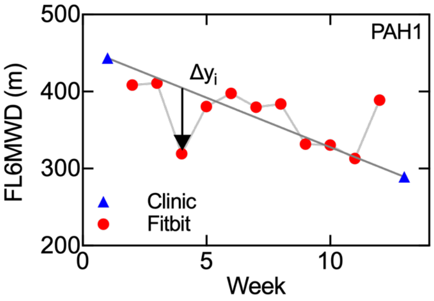

Supplementary Figure 25. The mean difference $\overline{\Delta y}$ between the interpolated clinic values (6MWD) and the FL6MWD was determined from the average of the weekly values Δy_i for each subject. (Triangles) clinic values, (circles) FL6MWD. The grey line shows the linear interpolation of the clinic values. To compare the FL6MWT to clinic values we used the following approach. Weekly "clinic" values were obtained from linear interpolation of the values at the two clinic visits at the beginning and end of the trial. We then calculated the difference between the FL6MWT and interpolated clinic value Δy_i for each week i. We then defined the mean value $\overline{\Delta y}$ for each subject. The set of mean values were then used as input to the k-means algorithm (Python 3.8, sklearn.cluster.KMeans). Using the silhouette method (Python 3.8, sklearn.metrics module), we identified two sub-groups: subjects with FL6MWT close to the interpolated clinic values (small $\overline{\Delta y}$, group 1: "performers"), and those with FL6MWT values below the interpolated clinic values (large $\overline{\Delta y}$, group 2: "underperformers"). The average value of $\overline{\Delta y}$ was –13.53 m for group 1 and -152.21 m for group 2.

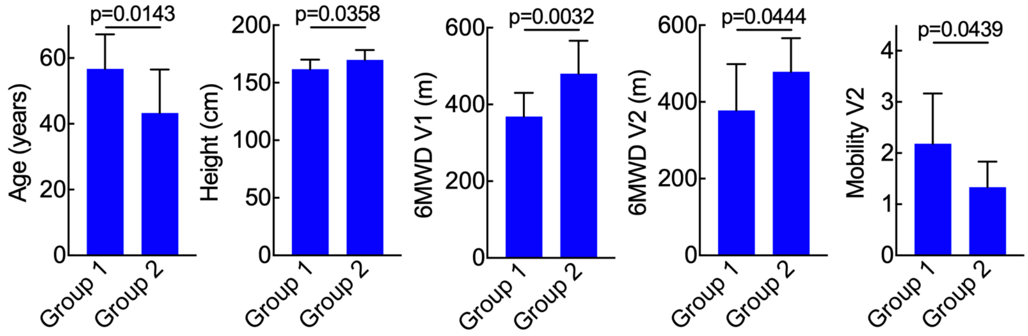

Supplementary Figure 26. Demographic and clinical parameters with statistical significance (Mann-Whitney test) between group 1 and group 2. Group 1: subjects with good agreement between interpolated clinic 6MWD and FL6MWD ("performers"), Group 2: subjects with values of FL6MWD consistently below the interpolated clinic values ("underperformers"). Bars represent mean ± SD.

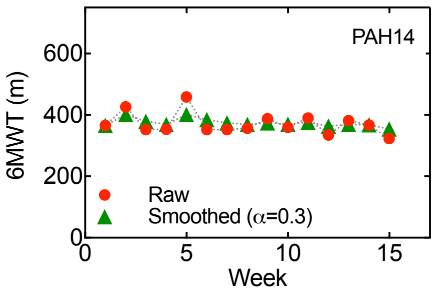

Supplementary Figure 27. Example of smoothing of FL6MWD. (Circles) raw data, (triangles) smoothed data. To assess changes in FL6MWT over time we performed a linear least squares fit following single exponential smoothing with $\alpha = 0.3$.

Supplementary Table 3. Bayesian Information Criteria (BIC) values for the multivariate ellipsoidal equal shape (VEV) model.

Number of clusters	BIC (VEV)
2	-1348.0
3	-1330.1
4	-1339.3

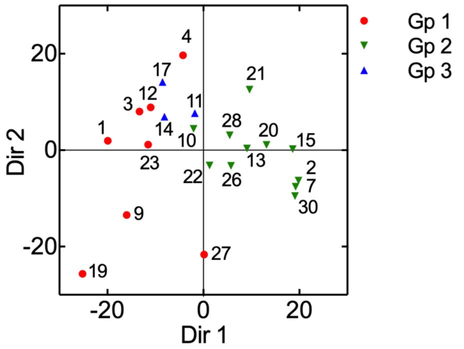

Supplementary Figure 28. Latent profile analysis using 8 Fitbit metrics. Numbers represent subject IDs. Latent profile analysis was performed using the ‘mclust’ package (version 5.4.10) in R (version 4.2.1). The optimal number of clusters was determined based on the maximum Bayesian Information Criterion (BIC) through the function ‘mclustBIC’. To visualize the clustering structure obtained from optimal model, a dimension reduction method was utilized via the function ‘MclustDR’ to project the data into the dimension reduced subspace. 8 Fitbit metrics were selected: daily step count, HR(SR=0), HR(SR=0)sk, HR(SR>0), ambulation product *P* value, fitness slope, FL6MWD, and usage. The highest BIC value (VEV model) was obtained for 3 groups. Group 1 (circles): PAH 1, 3, 4, 9, 12, 19, 23, 27. Group 2 (upside down triangles): PAH 2, 7, 10, 13, 15, 20, 21, 22, 26, 28, 30. Group 3 (triangles): PAH 11, 14, 17.

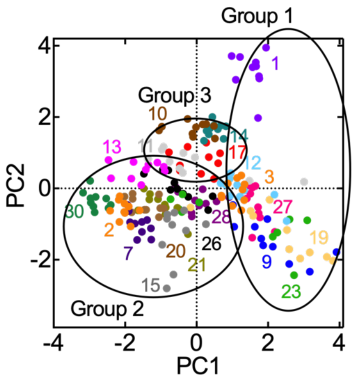

Supplementary Figure 29. Results from PCA analysis showing location of the three groups identified from LPA analysis. The three groups identified from LPA analysis occupy distinct regions of the PCA plot, with the exception of PAH 10 who is in Group 2. Numbers represent subject IDs. Each point represents one week of data.

Supplementary Table 4. Average values of Fitbit metrics within each group identified from LPA analysis.

	**Group 1**	**Group 2**	**Group 3**
n	8	11	3
Steps/Day	8024±2422	3966±1769	6076±504
HR(SR=0) (BPM)	83.4±15.8	75.2±7.4	89.2±3.1
HR(SR=0)sk	0.98±0.94	1.06±0.46	0.35±0.26
HR(SR>0) (BPM)	98.2±13.5	89.1±8.5	103.9±4.9
Ambulation product *P*	4033±3049	497±467	1169±980
Fitness slope	0.18±0.089	0.14±0.078	0.10±0.029
FL6MWD (m)	415±63	285±68	377±25
Usage	0.78±0.20	0.86±0.11	0.95±0.014

In general, Group 1 had high ambulation metrics (steps/day, ambulation product *P*, and FL6MWD), high HR(SR>0), and high fitness slope. Group 2 were characterized by the lowest ambulation metrics (steps/day, ambulation product *P*, FL6MWD), the lowest HR(SR=0) and HR(SR>0) (and highest HR(SR=0)sk). Group 3 had the highest HR(SR=0) and HR(SR>0), the lowest HR(SR=0)sk and fitness slope.

Supplementary References

1 Samson, M. M. *et al.* Differences in gait parameters at a preferred walking speed in healthy subjects due to age, height and body weight. *Aging Clin Exp Res* **13**, 16-21, doi:10.1007/Bf03351489 (2001).
