# Supplementary material for: Evaluation of physical health status beyond daily step count using a wearable activity sensor
Source: NPJ Digit Med. 2022 Nov 9;5:164. doi: 10.1038/s41746-022-00696-5 (PMC9646807; doi:10.1038/s41746-022-00696-5)
Supplement: Supplementary file 2 — Reporting Summary Checklist [file 41746_2022_696_MOESM2_ESM.pdf]

nature portfolio

Corresponding author(s): Peter SEarson

Last updated by author(s): Jul 31, 2022

Reporting Summary

Nature Portfolio wishes to improve the reproducibility of the work that we publish. This form provides structure for consistency and transparency in reporting. For further information on Nature Portfolio policies, see our Editorial Policies and the Editorial Policy Checklist.

Statistics

For all statistical analyses, confirm that the following items are present in the figure legend, table legend, main text, or Methods section.

n/a	Confirmed	
☐	☒	The exact sample size (*n*) for each experimental group/condition, given as a discrete number and unit of measurement
☒	☐	A statement on whether measurements were taken from distinct samples or whether the same sample was measured repeatedly
☐	☒	The statistical test(s) used AND whether they are one- or two-sided<br>*Only common tests should be described solely by name; describe more complex techniques in the Methods section.*
☐	☒	A description of all covariates tested
☐	☒	A description of any assumptions or corrections, such as tests of normality and adjustment for multiple comparisons
☐	☒	A full description of the statistical parameters including central tendency (e.g. means) or other basic estimates (e.g. regression coefficient) AND variation (e.g. standard deviation) or associated estimates of uncertainty (e.g. confidence intervals)
☐	☒	For null hypothesis testing, the test statistic (e.g. F, t, r) with confidence intervals, effect sizes, degrees of freedom and P value noted<br>*Give P values as exact values whenever suitable.*
☒	☐	For Bayesian analysis, information on the choice of priors and Markov chain Monte Carlo settings
☒	☐	For hierarchical and complex designs, identification of the appropriate level for tests and full reporting of outcomes
☐	☒	Estimates of effect sizes (e.g. Cohen's *d*, Pearson's *r*), indicating how they were calculated

Our web collection on statistics for biologists contains articles on many of the points above.

Software and code

Policy information about availability of computer code

Data collection	N/A
Data analysis	Matlab, R, Graphpad

For manuscripts utilizing custom algorithms or software that are central to the research but not yet described in published literature, software must be made available to editors and reviewers. We strongly encourage code deposition in a community repository (e.g. GitHub). See the Nature Portfolio guidelines for submitting code & software for further information.

Data

Policy information about availability of data

All manuscripts must include a data availability statement. This statement should provide the following information, where applicable:

- Accession codes, unique identifiers, or web links for publicly available datasets
- A description of any restrictions on data availability
- For clinical datasets or third party data, please ensure that the statement adheres to our policy

The data that support the findings of this prospective, observational study are not openly available but are available from the authors upon reasonable request. The data presented here are available within the article or supplementary materials.

Human research participants

Policy information about studies involving human research participants and Sex and Gender in Research.

Reporting on sex and gender	No analysis based on sex. Study size was too small
Population characteristics	22 individuals with PAH: 50.6±13.4 years old (mean±SD), with 3 males and 19 females.
Recruitment	Study details have been published elsewhere: S. Sehgal, A. Chowdhury, F. Rabih, A. Gadre, M. M. Park, M. S. Li, X. F. Wang, K. B. Highland and S.-C. Accelerometer. Counting Steps: A New Way to Monitor Patients with Pulmonary Arterial Hypertension. Lung 197, 501-508 (2019).
Ethics oversight	The study was approved by the IRB at the Cleveland Clinic (IRB number 15–1392).

Note that full information on the approval of the study protocol must also be provided in the manuscript.

Field-specific reporting

Please select the one below that is the best fit for your research. If you are not sure, read the appropriate sections before making your selection.

☐ Life sciences ☒ Behavioural & social sciences ☐ Ecological, evolutionary & environmental sciences

For a reference copy of the document with all sections, see nature.com/documents/nr-reporting-summary-flat.pdf

Behavioural & social sciences study design

All studies must disclose on these points even when the disclosure is negative.

Study description	Prospective, observational study of individuals with PAH.
Research sample	Individuals with PAH.
Sampling strategy	N/A
Data collection	Data were obtained from a prospective, observational study where PAH patients wore a wrist worn activity monitor (Fitbit Charge HR®) between two outpatient visits.
Timing	2/19/16 - 5/30/17
Data exclusions	22 of 30 data sets were included for analysis: these data sets had clinical data for both visits and at least 4 weeks of Fitbit data between the two visits. 3 subjects were excluded because they had no data on the Fitbit server, 3 subjects did not have a second clinic visit, and 2 subjects had only 1 week of Fitbit data.
Non-participation	Thirty subjects were enrolled in the study; two patients withdrew consent after enrollment.
Randomization	N/A

Reporting for specific materials, systems and methods

We require information from authors about some types of materials, experimental systems and methods used in many studies. Here, indicate whether each material, system or method listed is relevant to your study. If you are not sure if a list item applies to your research, read the appropriate section before selecting a response.

Materials & experimental systems

n/a	Involved in the study
☒	☐ Antibodies
☒	☐ Eukaryotic cell lines
☒	☐ Palaeontology and archaeology
☒	☐ Animals and other organisms
☒	☐ Clinical data
☒	☐ Dual use research of concern

Methods

n/a	Involved in the study
☒	☐ ChIP-seq
☒	☐ Flow cytometry
☒	☐ MRI-based neuroimaging
